# Oxidative Weathering and Microbial Diversity of an Inactive Seafloor Hydrothermal Sulfide Chimney

**DOI:** 10.3389/fmicb.2017.01378

**Published:** 2017-07-21

**Authors:** Jiangtao Li, Jiamei Cui, Qunhui Yang, Guojie Cui, Bingbing Wei, Zijun Wu, Yong Wang, Huaiyang Zhou

**Affiliations:** ^1^State Key Laboratory of Marine Geology, Tongji University Shanghai, China; ^2^Institute of Deep-Sea Science and Engineering, Chinese Academy of Sciences Sanya, China

**Keywords:** Fe-oxidizing bacteria, oxidative weathering, biomineralization, inactive hydrothermal chimney, microbial diversity, microbial succession

## Abstract

When its hydrothermal supply ceases, hydrothermal sulfide chimneys become inactive and commonly experience oxidative weathering on the seafloor. However, little is known about the oxidative weathering of inactive sulfide chimneys, nor about associated microbial community structures and their succession during this weathering process. In this work, an inactive sulfide chimney and a young chimney in the early sulfate stage of formation were collected from the Main Endeavor Field of the Juan de Fuca Ridge. To assess oxidative weathering, the ultrastructures of secondary alteration products accumulating on the chimney surface were examined and the presence of possible Fe-oxidizing bacteria (FeOB) was investigated. The results of ultrastructure observation revealed that FeOB-associated ultrastructures with indicative morphologies were abundantly present. Iron oxidizers primarily consisted of members closely related to *Gallionella* spp. and *Mariprofundus* spp., indicating Fe-oxidizing species likely promote the oxidative weathering of inactive sulfide chimneys. Abiotic accumulation of Fe-rich substances further indicates that oxidative weathering is a complex, dynamic process, alternately controlled by FeOB and by abiotic oxidization. Although hydrothermal fluid flow had ceased, inactive chimneys still accommodate an abundant and diverse microbiome whose microbial composition and metabolic potential dramatically differ from their counterparts at active vents. Bacterial lineages within current inactive chimney are dominated by members of α-, δ-, and γ-*Proteobacteria* and they are deduced to be closely involved in a diverse set of geochemical processes including iron oxidation, nitrogen fixation, ammonia oxidation and denitrification. At last, by examining microbial communities within hydrothermal chimneys at different formation stages, a general microbial community succession can be deduced from early formation stages of a sulfate chimney to actively mature sulfide structures, and then to the final inactive altered sulfide chimney. Our findings provide valuable insights into the microbe-involved oxidative weathering process and into microbial succession occurring at inactive hydrothermal sulfide chimney after high-temperature hydrothermal fluids have ceased venting.

## Introduction

Hydrothermal sulfide chimneys, typical products of seafloor hydrothermal activity, encompass a variety of complex and dynamic environments where hot, reductive hydrothermal fluid interacts with cold, oxygenated seawater. As one of the most productive ecosystems on Earth (Polz and Cavanaugh, 1995), active sulfide chimneys accommodate numerous unique microbial populations that obtain primary metabolic energy via chemosynthesis using a series of reactions made possible by the presence of intense chemical disequilibria between hydrothermal fluid and seawater. Since the discovery of seafloor hydrothermal vents (Corliss et al., 1979), many surveys have been performed to investigate microbial communities in active hydrothermal vent structures (Takai and Horikoshi, 1999; Hoek et al., 2003; Brazelton et al., 2006; Zhou et al., 2009; Anderson et al., 2015). Several recent analyses have further suggested that microbial community composition and distribution regularly shift in spatial distribution across active chimney walls in response to variable venting dynamics (Schrenk et al., 2003; Kormas et al., 2006; Li et al., 2014). When hydrothermal venting activity ceases, active sulfide chimneys no longer accumulate new sulfide deposits, and oxidative weathering becomes the dominant process. The steep thermal and chemical gradients that existed within active chimneys disappear, and a corresponding change of biotopes occurs (Erickson et al., 2009). A couple of studies suggest that microbial populations in inactive chimneys are remarkably distinct from those of actively venting structures, and that their biomass, diversity and activities are greater than those associated with active chimneys (Suzuki et al., 2004; Kato et al., 2010). Recently, Sylvan et al. (2012) revealed that a clear shift occurs from the dominant 𝜀*-Proteobacteria* and *Aquificae* in active sulfides to bacterial communities dominated by α-, β-, γ-, δ-*Proteobacteria* and *Bacteroidetes* in inactive hydrothermal sulfides, indicating that inactive chimneys are remarkably distinct microbial habitats. However, compared with numerous explorations of active hydrothermal structures, there have been few studies focusing on inactive chimneys and the process of microbial succession after active venting ceases. In addition, although it has been suggested that minerals of inactive chimneys serve as critical energy sources to support the metabolic growth of various chemolithoautotrophs (McCollom, 2000; Edwards et al., 2003a), little is known about the extent to which microbial communities associated with quiescent chimneys continue to utilize those energy sources.

After cessation of hydrothermal venting, inactive chimneys continuously interact with seawater under low-temperature conditions, resulting in the oxidative weathering and alteration of various metal sulfides. Earlier studies revealed that mineral-oxidizing bacteria directly use solid sulfide minerals as metabolic substrates and play a prominent role in the weathering of hydrothermal sulfide deposits (Wirsen et al., 1993; Eberhard et al., 1995; Edwards et al., 2003b). Textural observations provide unequivocal evidence that alteration and weathering products, i.e., Fe oxides, are mainly attributed to the biomineralization of autotrophic Fe-oxidizing bacteria (FeOB). However, microbiological evidence of the existence of Fe oxidizers is lacking (Edwards et al., 2003b). Fe-rich ultrastructures with characterized morphologies such as twisted stalks, indicative of certain known FeOB groups, can be produced by two taxonomically distinct species, raising the question as to what types of FeOB are present and contribute to oxidative weathering near hydrothermal vents (Kato et al., 2009; Li et al., 2012).

In the present study, an inactive sulfide chimney was collected from the Main Endeavor Field (MEF) of the Juan de Fuca Ridge (JDF) (Supplementary Figure S1). Yellow to brown alteration products had accumulated on the surfaces of this inactive chimney, indicating the occurrence of oxidative weathering of sulfide minerals. Mineral compositions, total organic carbon (TOC), and microbial community structures were determined at different spatial positions on this inactive sulfide chimney. Moreover, ultrastructures of surface-weathered products were also described to characterize microbial contributions during oxidative weathering. In addition, a young sulfate chimney, representing the primary stage of chimney formation, was collected from MEF and analyzed to identify the microbial inhabitants of this young chimney. Here, we present an examination of the oxidative weathering of an inactive sulfide chimney, we report correlations between Fe-oxidizing bacterial taxa and the products of alteration reactions, and we characterize microbial succession of hydrothermal chimneys at different stages.

## Materials and Methods

### Sampling and Description of Samples

**FIGURE 1:**
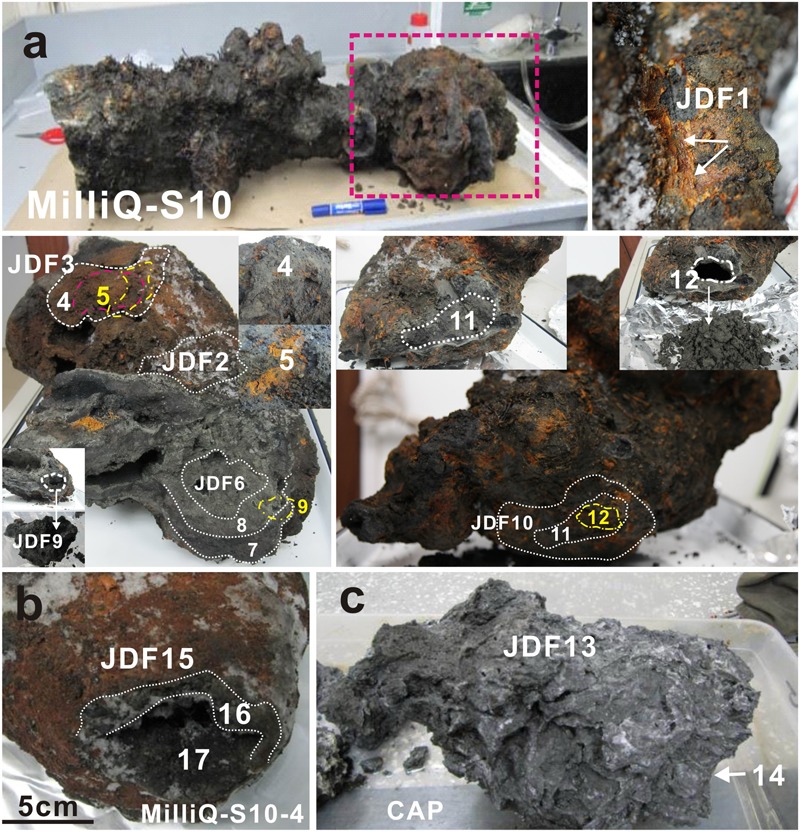
Positions of subsampling from the inactive sulfide chimney (Milli-Q S10 and Milli-Q S10-4) and young sulfate chimney (CAP). **(a)** Milli-Q S10 inactive sulfide chimney. Red rectangle indicates the analyzed segment in present study. JDF1 was stripped from yellow surficial chimney walls (shown by arrows). Both JDF2 and JDF3 were subsampled from the exterior walls but different locations. JDF4 was taken from the position just under JDF3, while JDF5 was obtained below JDF4 and it was a thin layer of microbial mats. JDF6–JDF8 were stripped step by step from the exterior to the interior around a fluid conduit. JDF9 was subsampled from the inner part of another conduit closely next to JDF6 (shown in figure). JDF10–JDF12 were also obtained layer by layer around one conduit at another side of the chimney. **(b)** Milli-Q S10-4 structure broken from the inactive chimney and spatial positions of subsampling JDF15–JDF17. JDF15 was stripped from the yellow outer walls, JDF16 was derived from the middle position, and JDF17 was subsampled from a site adjacent to the central channel. **(c)** Young active sulfate chimney CAP. JDF13 was collected from the exterior wall and JDF14 was taken from the middle part of chimney wall.

An inactive sulfide chimney and a young active sulfate chimney were collected from the MEF of the JDF during the AT18-08 cruise of the R/V *Atlantis* with the ROV *Jason II* (dive number J2-577) between July 19 and August 1, 2011 (Supplementary Figure S1). The inactive sulfide chimney, referred to as Milli-Q S10, was sampled from the Milli-Q site, and the young sulfate chimney, CAP, was obtained from the Dudley site (Supplementary Figure S1). Milli-Q S10 was approximately 1 m long with variable diameters from 15 to 50 cm and displayed several flange structures (Supplementary Figure S2). Generally, this chimney was dark overall but commonly coated with yellow and brown patches on its top portion (**Figure 1a**). There was no visible emission of fluid or colonization by living hydrothermal fauna. However, considerable numbers of dead tubeworms were attached to the surfaces of the Milli-Q S10 chimney (Supplementary Figure S2), supporting the inactivity of this chimney (Haymon et al., 2008). During the sampling by ROV *Jason II*, several small secondary structures were broken from the Milli-Q S10 chimney. One of these structures, referred to as Milli-Q S10-4, was cone shaped, with dimensions of approximately 20 cm in height, 15 cm in diameter at bottom and 10 cm in diameter on top (**Figure 1b**), exhibiting a thin reddish layer on the surface. Unlike Milli-Q S10, its surface was smooth, without any indications of tubeworms or other invertebrates. The active CAP chimney was collected from the top of a sulfide mound (Supplementary Figure S2). In 2006, cone-shaped cap equipment was placed on one active vent to monitor the formation of the chimney and microbial metabolic succession (Wang F. et al., 2009). After nearly 5 years, the initial small chimney had developed into a sulfide mound and our cap equipment was completely embedded within it. At the time of sampling, a newly formed sulfate chimney with a height of approximately 1.2 m was growing on the surface of the sulfide mound (Supplementary Figure S2). These chimneys were stored at -80°C immediately after shipboard recovery and preserved on dry ice during transportation to our lab, where they were stored at -80°C until further analyses.

### Subsampling from the Inactive Sulfide Chimney and Young Active Sulfate Chimney

The top section of the inactive sulfide chimney Milli-Q S10, which was spotted with yellow and brown secondary products and attached dead tubeworms (Supplementary Figure S2), was analyzed. Milli-Q S10 was an irregular chimney in which multiple, interconnected conduits of hydrothermal fluid were present. A total of 12 subsamples were collected from the exterior surfaces to the interior fluid conduits across the chimney walls, according to spatial positions within the chimney (**Figure 1a**). Four subsamples (JDF1-JDF3, JDF10) were collected from the exterior walls at different positions with different degrees of alteration or weathering, and three subsamples (JDF6, JDF9, and JDF12) were obtained from the interior walls close to the fluid conduits. For exterior samples, surface scrapings penetrated on average ∼2–3 mm. For interior samples, scrapings were taken ∼0–15 mm from the chimney exterior. Five other subsamples (JDF4, JDF5, JDF7, JDF8, and JDF11) were collected from the middle regions between the surfaces and interior conduitings. JDF5 consisted of a thin yellow layer considered as microbial mats which was yellow, soft and enriched in organic components (**Figure 1a**). Details of subsampling from Milli-Q S10 are shown in **Figure 1a**. By contrast, another inactive sulfide structure section, Milli-Q S10-4, which was broken from the Milli-Q S10 chimney, contained a single central conduit (**Figure 1b**). As shown in **Figure 1b**, three subsamples from the exterior surface (∼2–3 mm) (JDF15), middle layer (∼10–20 mm from the surface) (JDF16) and interior wall (∼0–10 mm from the exterior) (JDF17) were taken. The young sulfate chimney CAP was still active (Supplementary Figure S2), and many small pores and irregular channels were noted within its loose and fragile chimney walls (**Figure 1c**). Only two subsamples were collected from the outer (JDF13) and middle (∼5–8 cm from the surface) (JDF14) walls of this active chimney.

In addition, subsamples were collected for ultrastructure observation, including from the yellow weathered surficial chimney walls (WSS1, WSS3, and WSS15), microbial mats (JDF15) and mineralized tubes of dead tubeworms (T1, T2-L/S, T3-1) attached to the surficial walls of the inactive chimney.

### Mineralogical Analysis

Subsamples for mineralogical analysis were dried at 50°C and thoroughly ground to fine powder (∼0.074 mm) using a mortar and pestle. X-ray diffraction (XRD) pattern analysis was performed using a Rint 2000 X-ray diffractometer (Cu Kα radiation at 40 kV and 30 mA, scanning from 2 to 80°, scan velocity of 1° 2𝜃/min). Diffraction angles (referred to as ‘2𝜃’) corresponding to the atomic structure unique to each mineral were measured.

Subsample JDF1 obtained from the outmost yellowish surfaces of the inactive chimney was also subjected to Fourier transform infrared spectroscopy (FT-IR) because of its poor crystallinity. The FT-IR analysis was run with a Nciolet 5700 spectrometer using the KBr pellet method. Finely ground samples, ∼1 mg, were mixed with 200 mg KBr (FTIR grade, Fluka) in a vibratory ball mixer for 20 s. These mixtures were later pressed under a vacuum in a standard device at a pressure of 75 kN cm^2^ for 3 min to produce a transparent disk measuring approximately 1 mm in thickness and 13 mm in diameter. The spectral resolution was 4 cm^-1^, and the scanning range was from 600 to 4000 cm^-1^.

### Scanning Electron Microscopy (SEM)

Small fragments were collected from the inactive chimney, freeze dried thoroughly, and sputter coated with gold for 2–3 min. All were examined with a Philips XL-30 SEM instrument equipped with an EDAX energy-dispersive X-ray spectrometer and the corresponding analytical software. Energy-dispersive X-ray spectroscopy (EDS) was primarily qualitative in nature because of the irregular surface topography of the samples. The SEM was operated at 15 kV with a working distance of 10 mm to ensure optimum imaging and minimize charging and sample damage.

### Analysis of Total Organic Carbon (TOC)

Freeze-dried subsamples were homogenized with an agate mortar and pestle, then transferred into pre-weighed aluminum foil capsules, treated with 10% hydrochloric acid to remove carbonates, and dried overnight at 40–50°C. TOC values in hydrothermal precipitates were quantified with a Vario E1-III Elemental Analyzer interfaced with a Thermo Finnigan DELTA plus XL stable isotope ratio mass spectrometer. Analyses were normalized relative to an acetanilide standard. The concentrations of TOC were expressed as percent dry precipitates.

### DNA Extraction, Construction of Clone Libraries, Sequencing, and Phylogenetic Analyses

DNA was extracted using an SDS-based extraction method with some modifications (Zhou et al., 1996). In brief, approximately 5–10 g of precipitate was mixed with 13.5 mL of DNA extraction buffer (100 mM Tris-HCl, 100 mM sodium EDTA, 100 mM sodium phosphate, 1.5 M NaCl, 1% CTAB) and 100 μL of proteinase K (10 mg/mL, Sigma) in tubes via horizontal shaking at 225 rpm for 30 min at 37°C. After shaking, 1.5 mL of 20% (w/v) SDS was added, and the samples were incubated in a 65°C water bath for 2 h. The supernatants were collected after centrifugation at 6000 ×*g* for 10 min and transferred into 50 mL centrifuge tubes. The supernatants were mixed with an equal volume of chloroform isoamyl alcohol (24:1, v/v). The aqueous phase was recovered by centrifugation and precipitated with 0.6 volumes of isopropanol for approximately 1 h. Crude nucleic acids were obtained by centrifugation at 16,000 ×*g* for 20 min at room temperature, washed with 70% ethanol and resuspended in sterile deionized water. The crude nucleic acids were purified with the OMEGA EZNA^TM^ Cycle-Pure Kit.

Two subsamples, JDF5 and JDF15, which were obtained from microbial mats and the outmost chimney wall, respectively, were used to detect FeOB-associated species using 16S rRNA gene clone libraries. The bacterial 16S rRNA gene was amplified using the primers 27F (5′-AGA GTT TGA TCC TGG CTC AG-3′)/1492R (5′-GGT TAC CTT GTT ACG ACT T-3′) which produced ∼1500 bp products (Lane, 1991). The specific primers Zeta674F and 1492R were used to target members of ζ*-Proteobacteria* (McBeth et al., 2011), and the specific primers 122F (5′-ATA TCG GAA CAT GTC CGG-3′) and 998R (5′-CTC TGG AAA CTT CCT GAC-3′) were used to capture bacteria closely related to *Gallionella* spp. (Wang J. et al., 2009). Amplification conditions were consistent with the conditions listed in the references noted above. The PCR products were purified with a gel-extraction kit (Omega, United States) according to the manufacturer’s instructions. Purified PCR products were cloned into the pMD20-T vector (Takara, Japan) and transformed to competent *Escherichia coli* DH5α cells (Takara, Japan). The correct-size inserts were screened with the vector-specific primers M13f (5′-GTA AAA CGA CGG CCA G-3′) and M13r (5′-CAG GAA ACA GCT ATG AC-3′). Clones containing target 16S rDNA were selected randomly for sequencing with an ABI PRISM 3730XL automated sequencer with vector-specific universal primers. Bacterial 16S rRNA gene sequences were checked for chimeras using Bellerophon (3.0) at http://greengenes.lbl.gov (Huber et al., 2004). The DOTUR program was used to cluster sequences into operational taxonomic units (OTUs) or phylotypes using a 97% similarity cutoff (Schloss and Handelsman, 2005). All sequences and their close relatives obtained from GenBank and EMBL databases were aligned using ClustalX 2.0.1 (Larkin et al., 2007). FeOB-related sequences obtained from near full-length 16S rRNA gene sequences and other specific taxonomic targets together with sequences of known FeOB species were used to construct phylogenetic trees under maximum-likelihood criteria using Mega 5.0 (Tamura et al., 2011). Bootstrap analysis was used to provide confidence estimates of tree topologies.

### Pyrosequencing and Analysis of 16S rRNA Sequence Amplicons

DNA obtained from all 17 samples was quantified with a PicoGreen dsDNA Quantitation Kit (Life Technologies, Carlsbad, CA, United States). Total extracted DNA was used as template for PCR amplification of 16S rRNA genes using primers designed with Barcrawl (Frank, 2009). Bacterial and archaeal 16S rRNA genes were amplified using the primers 27F (5′-AGA GTT TGA TCC TGG CTC AG-3′)/533R (5′-TTA CCG CGG CTG CTG GCA C-3′) containing 10-nucleotide barcodes and Arch344F (5′-ACG GGG YGC AGC AGG CGC GA-3′)/Arch915R (5′-GTG CTC CCC CGC CAA TTC CT-3′) containing eight-nucleotide barcodes, respectively. The PCR reactions were first held in a thermocycler (Bio-Rad, Hercules, CA, United States) at 94°C for 5 min to denature the DNA, followed by amplification for 25 cycles at 94°C for 50 s, 53°C for 50 s, and 72°C for 50 s. A final extension of 6 min at 72°C was added to ensure complete amplification. The PCR products purified with the TaKaRa Agarose Gel DNA Purification Kit (TaKaRa, Dalian, China) were quantified using a NanoDrop ND-1000 device (NanoDrop, Wilmington, DE, United States) and sent for 454/Roche GS-FLX Titanium platform at Majorbio Bio-Pharm Technology, Co., Ltd., Shanghai, China.

Downstream analysis of the pyrosequenced amplicon reads was performed using QIIME 1.9.1. Reads of low quality were filtered out by enforcing the following quality control criteria: (1) exclusion of reads with one or more ambiguous nucleotides, (2) exclusion of reads shorter than 200 bp, (3) exclusion of reads containing homopolymers of 6 bp and above, and (4) exclusion of reads with an average flowgram score of 25 or below in a quality window of 50 bp. The quality-filtered reads were clustered into OTUs based on their sequence similarity, and a representative sequence from each OTU was selected using the longest picking method for downstream analysis. Taxonomy assignment was conducted using the RDP classifier against the SILVA 16S rRNA gene database (Version 119). Chimeric reads were identified and excluded using ChimeraSlayer in the QIIME package after alignment with PyNAST. Similarities among different microbial communities were determined using similarity matrices generated according to the phylogenetic distance between reads (Unifrac distance), and beta diversity of principal coordinates analysis (PCoA) was computed as components of the QIIME pipeline.

### Nucleotide Sequence Accession Numbers

The 16S rRNA gene sequences of FeOB-associated lineages reported in this paper were deposited in GenBank under accession numbers KX023409 – KX023416. The pyrosequencing data for 16S rRNA genes were deposited in the GenBank Short Read Archive (SRA) under accession ID SRP073405.

## Results

### Mineral Compositions

**FIGURE 2:**
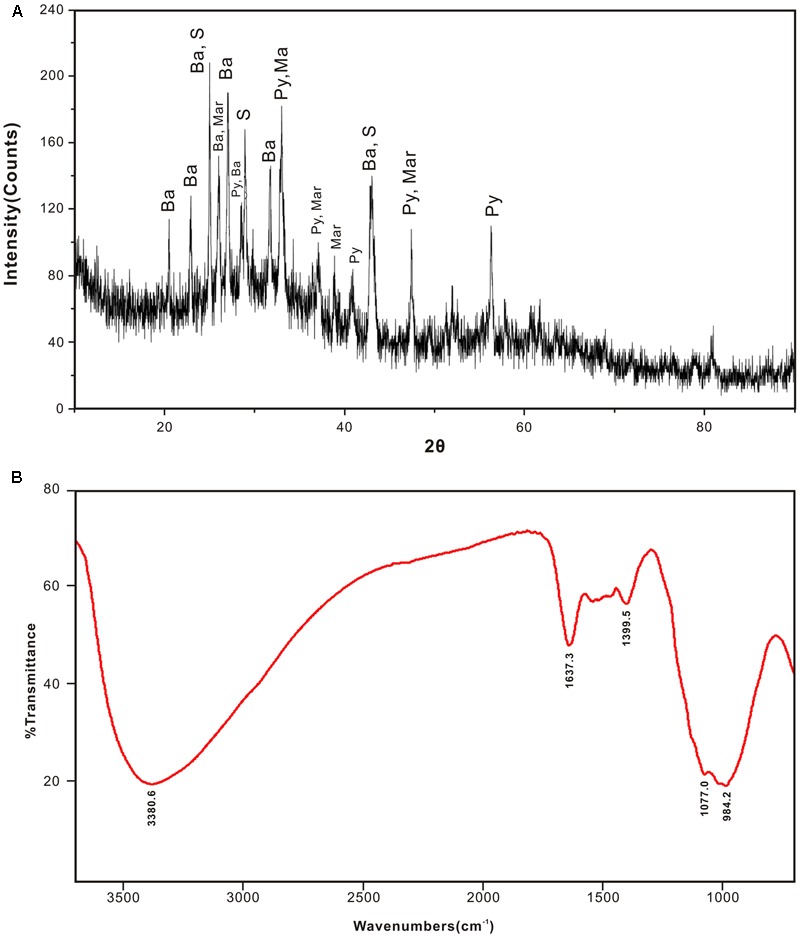
XRD pattern **(A)** and FTIR spectra **(B)** of JDF1 scratched from the outermost yellowish surface of inactive hydrothermal chimney, indicating that subsamples JDF1 mainly consist of amorphous mineral assemblages. The faint secondary peak at 2𝜃 of 61° and weak absorption peak at ∼1399 cm^-1^ indicate the presence of poor crystalline two-line ferrihydrites. In **(A)**, Ba: barite; S: sulfur; Py: pyrite; Mar: marcasite.

Mineral compositions and their abundances in different subsamples obtained from the inactive sulfide chimney and young active sulfate chimney were mainly determined using XRD analysis (**Table 1**). JDF1, scratched from the outmost yellowish surfaces of the inactive chimney, was subjected to XRD and FT-IR analyses because of its poor crystallinity. As shown in **Figure 2A**, the XRD pattern revealed that JDF1 mainly consisted of amorphous mineral assemblages and minor recognizable minerals including barite, pyrite, marcasite, and sulfur. The XRD pattern of amorphous mineral assemblages showed a broad hump at approximately 2𝜃 of 32° (d-spacing value ∼2.5 Å), and a very faint secondary peak at 2𝜃 of 61° (d-spacing value 1.5 Å) (**Figure 2A**), closely similar to that of poorly crystallized iron oxyhydroxide and indicating the presence of two-line ferrihydrite (Boyd and Scott, 1999; Kennedy et al., 2003). FT-IR spectra also provided valuable information about Fe oxyhydroxides (**Figure 2B**). There were several prominent bands at ∼ 3380, 1637, 1399, 1077, and 984 cm^-1^. The band at 3380 cm^-1^ was assigned to the stretching vibration of -OH from surface H_2_O molecules or from an envelope of hydrogen-bonded surface -OH groups (Koji and Solomon, 1977; Gotić and Musić, 2007). The band at 1637 cm^-1^ was close to the position of H_2_O bending vibrations (Ryskin, 1974). The relatively weak peak, which appeared at ∼1399 cm^-1^, was assigned to Fe-OH (Tuysuz et al., 2008), suggesting the presence of ferrihydrite. The bands at ∼980–1100 cm^-1^ could be attributed to the Si-O stretching vibration from the SiO_4_ tetrahedra with three or four bridge O atoms (Farmer, 1974; Vempati et al., 1990). Therefore, it was inferred from XRD and FT-IR analyses that JDF1, derived from yellowish substrates of the outermost surfaces of inactive chimney walls, mainly consisted of amorphous Fe oxyhydroxides. XRD recognizable minerals in JDF1 most likely came from the underlying mineral assemblages when we scratched the thin surfaces to sample (**Figure 1**). JDF5, identified as microbial mats (**Figure 1a**), was not analyzed because of its low masses.

**Table 1:** Mineral compositions (wt%) of different subsamples taken from different spatial positions of the inactive sulfide chimney and an active young sulfate chimney.

Spatial positions of subsamples	No. of chimneys	Subsamples^∗^	Sphalerite	Wurtzite	Pyrite	Marcasite	Chalcopyrite	CuS	Barite	Anhydrite	Gypsum
Exterior walls	Milli-Q S10	JDF1	Mainly composed of amorphous Fe oxyhydroxides such as ferrihydrites with minor barite, marcasite, pyrite, and sufur.							
		JDF2	55.5	15.0	17.9	nd	nd	11.1	minor	nd	nd
		JDF3	32.8	10.0	19.7	19.2	nd	8.2	10.1	nd	nd
		JDF7	46.0	nd	22.7	24.0	nd	nd	7.3	nd	nd
		JDF10	48.1	nd	15.3	29.3	nd	nd	7.4	nd	nd
	Milli-Q S10-4	JDF15	57	5.7	16.4	7.5	nd	6.7	6.6	nd	nd
	CAP	JDF13	nd	nd	nd	nd	nd	nd	nd	95.0	5.0

Middle layers	Milli-Q S10	JDF4	67.9	7.0	20.0	nd	nd	5.1	2.7	nd	nd
		JDF8	49.6	4.3	18.8	19.4	3.0	nd	4.9	nd	nd
		JDF9	42.2	minor	14.3	29.7	nd	nd	13.7	nd	nd
		JDF11	87.1	nd	12.9	nd	nd	nd	nd	nd	nd
		JDF5	Identified as microbial mats and no XRD analysis was determined because of its low masses							
	Milli-Q S10-4	JDF16	77.7	nd	22.3	nd	nd	nd	minor	nd	nd
	CAP	JDF14	nd	1.2	nd	nd	nd	nd	nd	92.5	6.3

Inner parts	Milli-Q S10	JDF6	34.5	nd	33.0	7.3	22.1	nd	3.1	nd	nd
		JDF12	59.8	3.9	32.5	nd	0.7	nd	minor	nd	nd
	Milli-Q S10-4	JDF17	82.6		17.3	nd	nd	nd	minor	nd	nd

*^∗^‘nd’ means not detected; ‘minor’ means mineral compositions are <1%.*

Except JDF1, other subsamples of Milli-Q S10 chimney and the S10-4 structure primarily consisted of various sulfide minerals such as sphalerite, wurtzite, pyrite, marcasite, and chalcopyrite (**Table 1**). Among them, sphalerite and pyrite were the most common and abundant mineral assemblages in all subsamples with contents of 32.8–82.6 and 12.9–33.0 wt%, respectively. Although contents of marcasite were sometimes high, varying from 7.3 to 29.7 wt%, their distributions were limited. Wurtzite was generally less than 15 wt%. Chalcopyrite was found only in locations closely adjacent to the central fluid channels such as JDF6, JDF8, and JDF12 and its abundance could be up to 22.1 wt%. By contrast, the active young CAP chimney was distinct in mineral compositions from those of the inactive sulfide chimney and structure. Two subsamples of the CAP chimney predominantly consisted of anhydrite (92.5–95.0 wt%) with minor gypsum (5.0–6.3 wt%). Only a small amount of wurtzite (1.2 wt%) was detected from JDF14.

### Total Organic Carbon (TOC) and δ^13^C_TOC_

**FIGURE 3:**
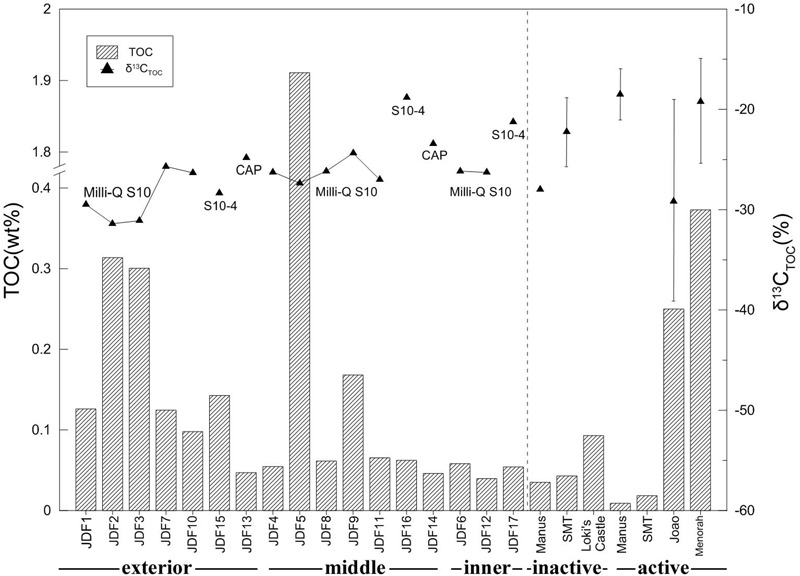
Total organic carbon (TOC) and stable carbon isotopic ratio of subsamples collected from different spatial positions of an inactive sulfide chimney and an active sulfate chimney. For comparison with TOC contents and δ^13^C_TOC_ values, data of inactive and active chimneys from Manus back-arc basin (Reeves et al., 2014), Loki’s Castle of the Arctic Mid-Ocean Ridge (Joao and Menorah) (Jaeschke et al., 2012) and the Southern Mariana Trough (SMT) (Kato et al., 2010) are also plotted together (the vertical lines indicating the ranges of δ^13^C_TOC_ values and triangles representing the mean values).

Total organic carbon concentrations varied from 0.04 to 1.91 wt% (**Figure 3**). The highest TOC concentration (1.91 wt%) was detected in the microbial mats of JDF5. Generally, TOC content decreased from the exterior positions to the inner positions across the inactive chimney walls (**Figure 3**). For example, within Milli-Q S10, TOC concentrations of several outer subsamples were commonly greater than 0.10 wt%, especially in JDF2 and JDF3, whose TOC values reached 0.30 wt%. In sharp contrast, two interior subsamples, JDF6 and JDF12, contained only 0.06 and 0.04 wt% TOC concentrations, respectively. The TOC concentrations of subsamples collected from the middle layers fell between those of the exterior and inner regions, with a range of 0.06–0.17 wt%. A similar trend was present within the Milli-Q S10-4 structure (**Figure 3**). Two subsamples from the young CAP chimney had comparatively low TOC contents of approximately 0.05 and 0.06 wt%.

The values of δ^13^C_TOC_ ranged from -18.82 to -31.39‰ (**Figure 3**). Within the subsamples of Milli-Q S10, the lowest values of δ^13^C_TOC_ were measured from two surficial subsamples, JDF2 and JDF3, with values of -31.39 and -31.10‰, respectively. Other subsamples from middle and inner regions displayed similar δ^13^C_TOC_ values varying between -27.02 and -24.36‰ (**Figure 3**). For the subsamples of structure Milli-Q S10-4, although the exterior JDF15 had δ^13^C_TOC_ value of -28.32‰ comparable to those of Milli-Q S10, the middle JDF16 and the inner JDF17 possessed the heaviest δ^13^C_TOC_ with values of -18.82 and -21.24‰, respectively, yielding a difference of ∼10‰ (**Figure 3**). JDF13 and JDF14, subsamples of the active CAP sulfate chimney, possessed δ^13^C_TOC_ values of -24.84 and -23.44‰, respectively.

### Morphological Observation and Description of Ultrastructures

**FIGURE 4:**
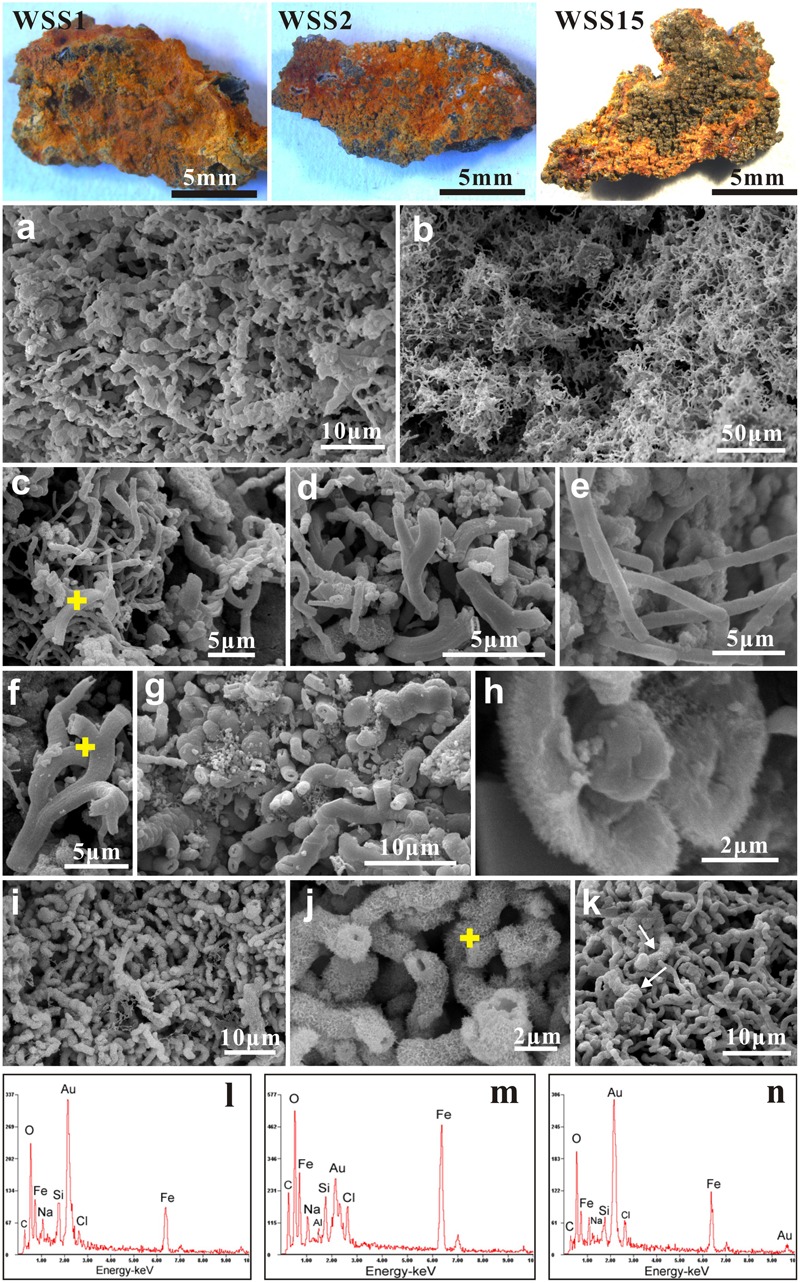
Scanning electron microscopy (SEM) images of weathered inactive sulfide chimney surfaces (WSS1, WSS3, and WSS15). **(a,b)** Dense aggregates of FeOB-associated ultrastructures such as twisted ribbon-like stalks, branched stalks, straight tubular sheaths and contorted filaments. **(c–f)** Typical morphologies of Fe-rich ultrastructures induced by known FeOB species. Yellow crosses indicated the positions for EDS analyses. **(g)** Locally, some branched stalks are heavily encrusted by Fe precipitates. **(h)** Magnified images of stalk surfaces with tiny needlelike textures. **(i,j)** Stalks encrusted by bladelike Fe precipitates. Chemical compositions of bladelike textures (yellow cross) was analyzed with EDS. **(k)** Common occurrence of freshly biogenic stalks and “modified” stalks by abiogenic Fe precipitates. **(l,m)** EDS spectra of FeOB-associated stalks marked with yellow cross in **(c,f)**, respectively. Iron was the dominant chemical compositions (>50 wt%), followed by variable amounts of C and Si. **(n)** Chemical contents of needle-like and flower-like architectures in **(j)** which are mainly composed of Fe.

**FIGURE 5:**
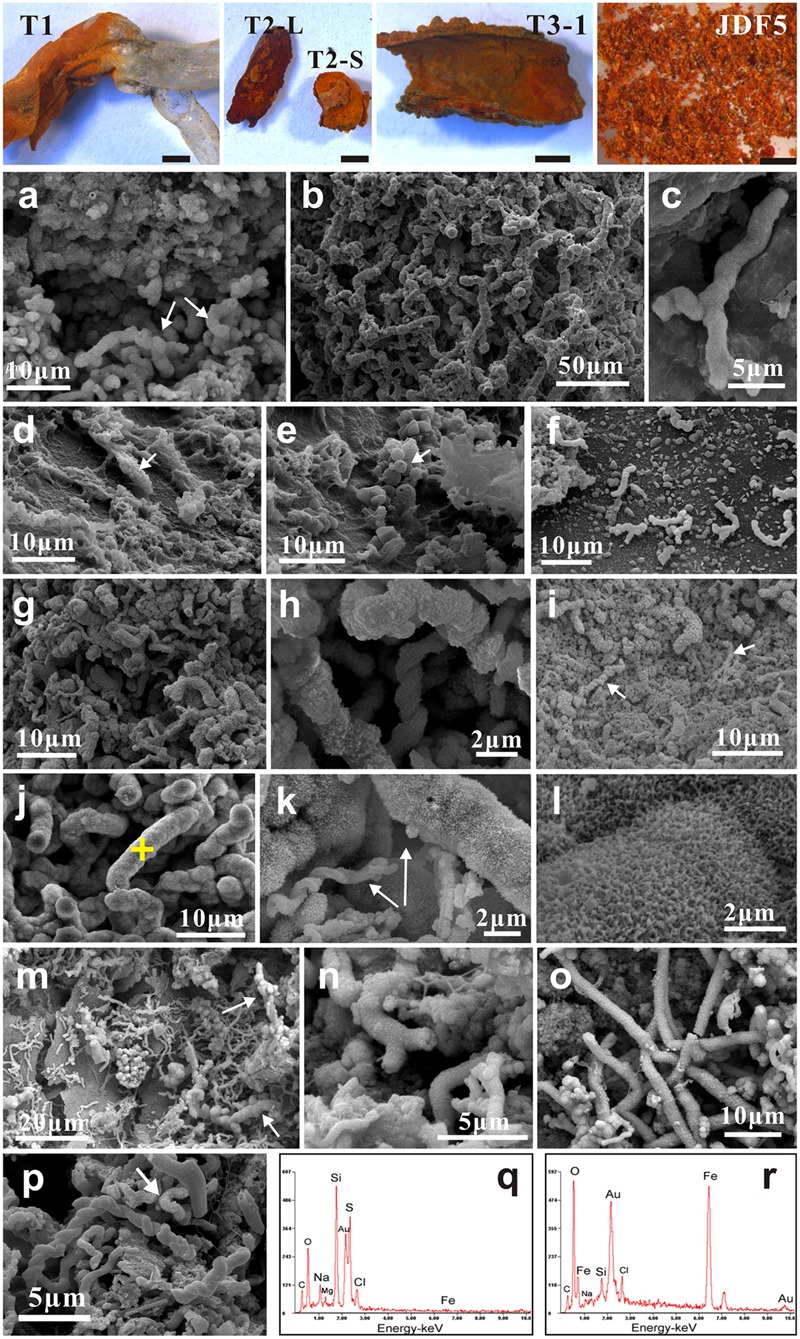
Characteristics of ultrastructures detected from mineralized and fossilized tubes of dead tubeworms, and microbial mats. **(a–f)** T1 tubeworm. The yellow part contains abundant FeOB-associated stalks and filaments heavily aggregated by tiny Fe-rich flakes **(a–c)**. However, structures produced by FeOB are sporadically scattered in the gray part **(d–f)**. Alternatively, irregularly or spherically S-enriched aggregates (arrows in **d,e**) are prevailing. **(g–l)** T2-L and T2-S tubeworms. T2-L’s stalks **(g–i)** are heavily covered by irregularly tiny granules **(h)** and often buried into amorphous Fe-rich substances **(i)**. T2-S’s structures **(j–l)** are mainly dominated by contorted filaments. Magnified images show that these filaments have acanthaceous surfaces **(k,l)** and they are remarkably thicker than fresh stalks (arrows). **(m–o)** Typical ultrastructures detected from the fossilized tube wall of T3-1. Abiogenic amorphous Fe-rich precipitations significantly obscure the skeletons of these structures and also thicken them in diameters (**m**, arrows). **(p)** Fresh FeOB-associated twisted stalks and structures heavily encrusted by bladelike Fe-rich aggregates (arrow). **(q)** EDS chemical composition of spherical aggregates in **(e)**, indicating they are enriched in sulfur. **(r)** EDS spectra of acanthaceous surfaces marked by yellow cross in **(j)**, mainly composed of iron.

Numerous biogenic ultrastructures with distinctive morphologies were detected from the weathered surficial inactive chimney walls (**Figure 4**), the mineralized tubes of dead tubeworms (**Figure 5**), and microbial mats (**Figure 5p** and Supplementary Figure S3). Among them, several kinds of distinguishable ultrastructures including twisted ribbon-like stalks (**Figures 4a, 5p**), branched stalks (**Figures 4d,f** and Supplementary Figure S3), straight tubular sheaths (**Figures 4e, 5o**) and irregular filaments (**Figures 4b, 5m**) were dominantly abundant and generally occurred in massive accumulations (**Figures 4a, 5a** and Supplementary Figure S3). They commonly showed a hollow tubular aspect 0.5–2 μm in diameter and up to tens of μm in length (**Figures 4a–g** and Supplementary Figure S3). EDS analyses revealed that these ultrastructures were primarily composed of Fe, commonly more than 50 wt%, followed by variable amounts of C and Si (**Figures 4l,m**). All these characteristics of morphologies and chemical components of ultrastructures (ribbon-like stalks, branched stalks, straight tubular sheaths, and irregular filaments) firmly demonstrated that they were typically biogenic FeOB-associated ultrastructures which are the exclusive products of several known Fe-oxidizing species such as *Mariprofundus ferrooxydans, Gallionella ferruginea*, and *Leptothrix ochracea* (Emerson et al., 2010; Chan et al., 2011; McAllister et al., 2011; Krepski et al., 2013). In most cases, these FeOB-associated ultrastructures had smooth surfaces (**Figures 4a–f**) similar to the surfaces of freshly produced stalks by FeOB species (Chan et al., 2011).

A considerable number of ultrastructures were heavily encrusted with striking surficial morphological architectures such as needle-like architectures (**Figures 4g,h**), thin nanosheets (**Figures 5a,c**), irregularly tiny grains (**Figures 5g,h**) and fine burrs (**Figures 5j–l**). Some thin nanosheets further conglomerated closely and formed flower-like aggregates (**Figures 4i–k**). EDS analyses showed these surficial architectures were mainly composed of iron (**Figures 4n, 5r**). These needle-, nanosheet-, bur- and flower-like architectures were ascribed to typical textures of ferrihydrites formed by abiotic chemical Fe^2+^ oxidization (Zhang et al., 2014). In addition, these modified FeOB-associated ultrastructures also showed a broader diameter range than those from fresh ones, varying from approximately 0.5 μm to nearly 5.0 μm. Formless agglomerates, including globules, flaky aggregates, irregularly shaped blebs and amorphous materials, were common and were primarily attributed to Fe oxyhydroxides/oxides that were precipitated though the abiotic oxidization of Fe^2+^.

In addition, differences were observed in terms of microbial architectures among different mineralized tubeworm tubes or even different portions of the same tube. Within the T-1 tube, yellowish and gray regions showed distinctive ultrastructures (**Figures 5a–f**). The yellowish portion contained abundant biogenic stalks and filaments with different degrees of mineralization, whereas the gray portion was dominated by many formless aggregates originated from abiotic precipitation, with FeOB-related textures sporadically observed. Moreover, coccoidal forms and their aggregates (**Figures 5d,e**), primarily composed of sulfur (**Figure 5q**), were detected only in the gray portion, which indicated the possible occurrence of S-oxidizing microbial groups. Tubular sheaths, whose frequency of occurrence was low in most subsamples of chimney and tubes, were fairly abundant in T3-1 tubes (**Figure 5o**), whereas heavily mineralized contorted filaments prevailed within the T2-S tube (**Figure 5j**).

### Microbial Community Structures Revealed by 16S rRNA Gene Pyrosequencing

**FIGURE 6:**
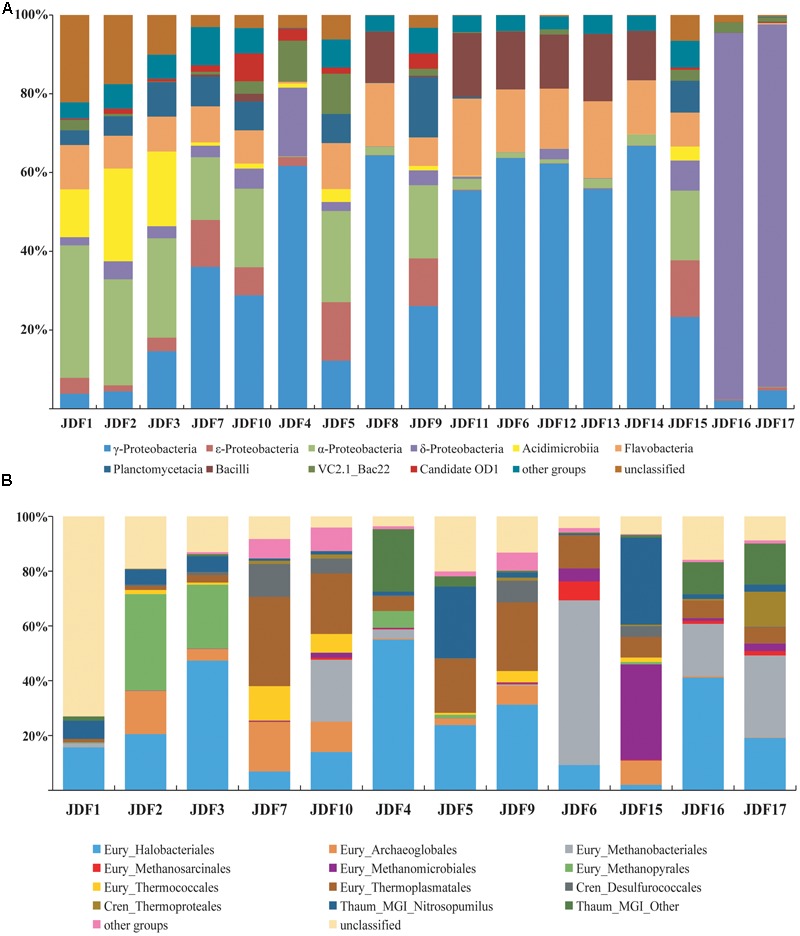
Compositions of the bacterial **(A)** and archaeal **(B)** communities based on the 16S rRNA genes for each subsample of inactive sulfide chimney and young sulfate chimney. Their relative abundances are also shown. Members which made up less 5% of reads were grouped into other groups.

A total of 183,410 tags of the bacterial 16S rRNA gene for 17 subsamples were collected from the inactive Milli-Q S10, Milli-Q S10-4, and active young CAP chimneys, and these tags were further clustered into 4,778 OTUs using a 97% similarity cutoff (Supplementary Table S1). Phylogenetic analysis showed that at the class level, bacterial OTUs were primarily classified into α-, γ-, 𝜀-, and δ-*Proteobacteria*; *Bacilli* of the phylum *Firmicutes*; *Planctomycetacia* of the *Planctomycete*s; *Flavobacteria* and uncultured VC2.1_Bac 22 within the *Bacteroidetes*; and *Acidimicrobiia* of the phylum *Actinobacteria* (**Figure 6A**).

Signatures of δ*-Proteobacteria* predominantly accounted for more than 90% of the reads from subsamples JDF16 and JDF17, which had similar community structures; by contrast, within other subsamples, the abundance of δ*-Proteobacteria* was generally less than 5% (**Figure 6A**). Moreover, most signatures detected from JDF16 and JDF17 were exclusively assigned to the order *Bdellovibrionales* (>90%) (Supplementary Table S1). Currently, little is known about this lineage (Yilmaz et al., 2016), and it has been suggested that members of this order are primarily found in the epipelagic zone of tropical waters in lower latitudes (DeLong et al., 2006; Pham et al., 2008; Yilmaz et al., 2012). Recently, general molecular surveys of marine bacterial communities have also identified abundant *Bdellovibrionales*-related 16S rRNA gene sequences from deep-sea inactive or low-temperature vents (Sylvan et al., 2012; Meyer et al., 2013). Signatures of γ*-Proteobacteria* were among the most common within all samples, and in most cases, they accounted for 23% to nearly 67% of the bacterial community. The majority of γ*-Proteobacteria* further fell into the genera *Pseudomonas* and *Nitrosococcus* (Supplementary Table S1). Signatures of α*-Proteobacteria* were also common and relatively abundant. Especially in several outer samples, these components were predominant and accounted for nearly 25–34% of bacterial compositions (**Figure 6A**). These members were primarily scattered within the orders *Rhizobiales* and *Rhodobacterales* (Supplementary Table S1). Pyrotags affiliating with *Bacteroidetes* were detected in all inactive sulfide subsamples with relatively uniform and stable abundances ranging between 10 and 21% of libraries and accounting for less than 3% of tags within two young sulfate subsamples (Supplementary Table S1). As shown in **Figure 6A**, most of the *Bacteroidetes* signatures belonged to the class *Flavobacteria*. The *Firmicutes*, whose signatures came from the class *Bacilli*, were derived from four sulfide samples (JDF6, JDF8, JDF11, and JDF12) and from the young sulfate chimney samples (JDF13 and JDF14) and contributed approximately 13–18% of bacterial pyrotags within these samples (Supplementary Table S1). Other taxonomic groups, such as, *Actinobacteria*, 𝜀*-Proteobacteria*, and *Planctomycetes* were also common in these subsamples, but their distributions were significantly heterogeneous (**Figure 6A**). Fe-oxidizing bacterial groups mainly belonged to the genus *Mariprofundus* of ζ*-Proteobacteria* and the family *Gallionellaceae* of β*-Proteobacteria* (Supplementary Table S1). *Mariprofundus*-associated groups were detected in 10 out of the 17 subsamples, and especially in those subsamples from chimney exterior walls and microbial mats (JDF5), their proportions could reach up to 3.5% (Supplementary Table S1). By contrast, signatures of FeOB members of the *Gallionellaceae* were detected only from JDF15 where they represented ∼0.3% of bacterial reads. In addition, a subset of sequence tags belonged to unclassified bacterial clades, suggesting the existence of unknown bacterial populations (**Figure 6A**).

A total of 73,012 sequence tags of archaeal 16S rRNA genes were obtained for 12 subsamples of the Milli-Q S10 and Milli-Q S10-4 inactive chimneys (Supplementary Table S2). We failed to amplify archaeal sequences from three inactive sulfide subsamples (JDF8, JDF11, and JDF12) of Milli-Q S10 or from two subsamples (JDF13 and JDF14) of the young CAP chimney. Archaea dwelling in inactive chimneys were primarily composed of members of the phyla *Euryarchaeota, Thaumarchaeota*, and *Crenarchaeota* (**Figure 6B**). The *Korarchaeota, Nanoarchaeota* and Marine Hydrothermal Vent Group-1 (MHVG-1), which is one of the deepest-branching archaeal lineages (Takai and Sako, 1999), were also detected but in low abundance (Supplementary Table S2).

The diversity of the phylum *Euryarchaeota* was high and contained a series of archaeal groups with different abundances and heterogeneous distributions among subsamples. Signatures of the order *Halobacteriales* were among the most abundant archaeal populations in these 12 subsamples and in several subsamples their abundance varied from 31 to 55% (**Figure 6B**). The majority of these sequences were derived from the Deep-sea Hydrothermal Vent Euryarchaeota Group 6 (DHVEG-6) (Takai and Horikoshi, 1999), which is closely related to the *Candidatus* “Parvarchaeum” (Baker et al., 2010) (Supplementary Table S2). Organisms belonging to DHVEG-6 are primarily associated with deep-sea hydrothermal vent systems (Takai and Horikoshi, 1999; Teske and Sørensen, 2008; Li et al., 2014) but have also been found in marine sediment, hypersaline water, fresh water, and anoxic soils (Hugoni et al., 2015). Other minor lineages within the *Halobacteriales* included the *Halobacteriaceae*, Deep Sea Euryarchaeotic Group (DSEG), Marine Hydrothermal Vent Group (MHVG), and Miscellaneous Euryarchaeotic Group (MEG) (Supplementary Table S2). The order *Thermoplasmatales* was also commonly detected in these 12 subsamples but with variable abundances. Within several samples, such as JDF5, JDF7, JDF9 and JDF10, these groups contribute to 20–33% of reads (**Figure 6B**). Most of these tags could be further ascribed to the Deep-sea Hydrothermal Vent Euryarchaeotic Group 2 (DHVEG-2), which is a common and important clade of the microbial community in vent environments (Supplementary Table S2) (Reysenbach et al., 2006). In addition, within the *Euryarchaeota*, several archaeal lineages were detected that were affiliated with taxa known to be involved in methane cycling including the orders *Methanococcales, Methanobacteriales, Methanosarcinales, Methanomicrobiales*, and *Methanopyrales* (**Figure 6B**), and their relative abundances and distributions were highly variable among different samples. For example, *Methanomicrobiales* was abundant in only JDF15 where it comprised almost 35% of reads, whereas signatures of *Methanopyrales* were abundantly in JDF2 and JDF3, ranging from 23 to 35% of archaeal reads (**Figure 6B**). The group for which the highest abundance of signatures was recovered, *Methanobacteriales*, was detected in JDF6, JDF10, and JDF16-JDF17. In JDF6, 60% of reads were affiliated to this group (**Figure 6**). In addition, the remainder of Euryarchaeotic lineages, such as the orders *Methanococcales, Methanosarcinales, Archaeoglobales* and *Thermococcales*, appeared to be in low abundance and contributed minimally to overall archaeal profiles in most samples (Supplementary Table S2). The phylum *Crenarchaeota* was primarily composed of members of *Desulfurococcales* and *Thermoproteales*. Although these groups were found in most samples, their abundances were generally not high. As shown in **Figure 6B**, members of the phylum *Thaumarchaeota* were also common, and in certain samples, they consisted of ∼20–33% of the archaeal communities. Their subclades consisted of the Marine Group I (MGI), Marine Benthic Group A (MBG-A), MBG-B, Miscellaneous Crenarchaeotic Group (MCG), Soil Crenarchaeotic Group (SCG), South African Gold Mine Group 1 (SAGMCG-1), Terrestrial Hot Spring Group (THSCG) and pMC2A209 (Supplementary Table S2). Most of the members occurred in the MGI clade (**Figure 6** and Supplementary Table S2).

### Fe-Oxidizing Bacteria (FeOB)-Associated Bacterial Communities Based on the Construction of 16S rRNA Gene Clone Libraries

**FIGURE 7:**
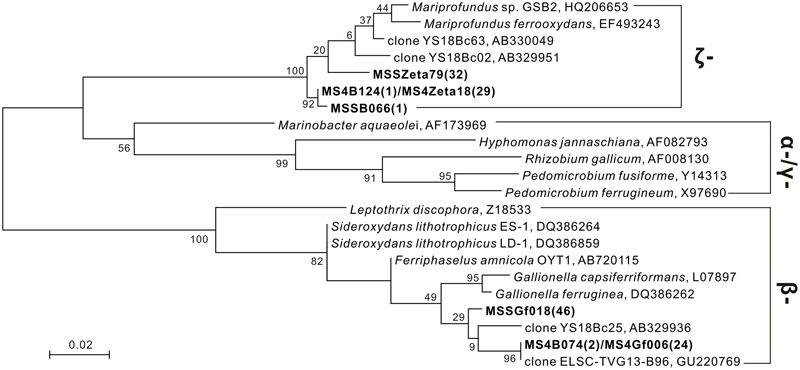
Phylogenetic tree under maximum likelihood criteria of FeOB-associated OTUs (bold) detected from our present study together with other known iron-oxidizing bacterial species and environmental clones (Emerson and Moyer, 2002; Kato et al., 2009, 2014; Li et al., 2012). Sequences of 16S rRNA gene obtained from universal bacterial primers 21F/1492R (∼1.5 kb) and FeOB-specific primers Zeta674F/1492R (800 bp), 122F/998R (900 bp) are combined together to construct the phylogenetic tree. Numbers in brackets indicate the number of clones. Bootstrap support is shown at nodes for 1000 bootstrap replicates under maximum likelihood.

Several sequences closely related to known iron oxidizers were obtained from the 16S rRNA gene clone library (**Figure 7**). With universal bacterial primers 21F and 1492R (Lane, 1991), 80 and 104 clones were obtained from subsamples JDF1 and JDF5, respectively, and these clones were classified into 48 and 71 OTUs based on 97% similarity cutoff, respectively. Only two OTUs, MSSB066 and MS4B124 (each representing 1 clone), were detected from JDF1 and JDF5, that showed high similarities to *Mariprofundus* spp., and were ascribed to the class ζ*-Proteobacteria* (**Figure 7**). Another OTU, MS4B074, which represented 2 clones and was closely related to *Gallionella* spp. affiliated with the class β*-Proteobacteria*, was detected from JDF5 (**Figure 7**).

Specific primers Zeta674F and 1492R were used to target members within the ζ*-Proteobacteria* (McBeth et al., 2011). 29 and 32 clones were obtained from subsamples JDF1 and JDF5, respectively, and they were clustered into two OTUs, MS4Zeta18 and MSSZeta79. These two OTUs showed close identity (95–96%) to *M. ferrooxydans* (**Figure 7**). MS4Zeta18 was determined to be the same OTU as MS4B124 (above) based on a 97% similarity cutoff of the 16S rRNA gene. Additionally, to capture microbes closely related to *Gallionella* spp., specific primers 122F and 998R were used (Wang J. et al., 2009). 24 and 46 clones were obtained from subsamples JDF1 and JDF5, respectively, and they were clustered into two OTUs, MSSGf006 and MSSGf018. MS4Gf006 was identified as the same OTU as MS4B074 (**Figure 7**), and they were also nearly identical to two environmental sequences YS18Bc63 and ELSC-TVG13-B96 from the hydrothermal niches of Mariana Trough (Kato et al., 2009) and Lau Basin (Li et al., 2012), respectively, which were classified into the genus *Gallionella* with >99% sequence similarity.

### Statistical Results of Principal Coordinates Analysis (PCoA)

**FIGURE 8:**
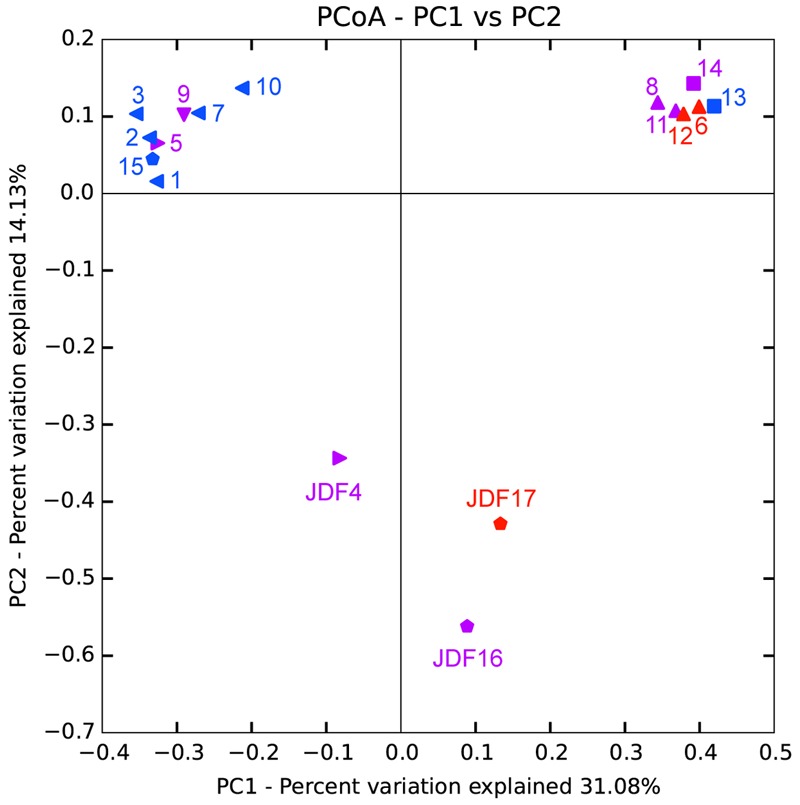
Principal component analysis (PCoA) of bacterial 16S rRNA gene sequences obtained from different spatial positions of inactive sulfide chimneys and active young sulfate chimney. ***Triangle:*** subsamples from Milli-Q S10; ***Pentagen:*** subsamples from Milli-Q S10-4; ***Square:*** subsamples from CAP. ***Red***: subsamples from inner parts; ***Purple***: subsamples from middle layers; ***Blue***: subsamples from exterior walls.

Bacterial and archaeal 16S rRNA gene sequences obtained from pyrosequencing from different chimney parts were subjected to PCoA. As shown in **Figure 8**, PCoA results for bacterial tag sequences showed that the first two principal components (PC1 and PC2) explained 45.21% of the total variation and 17 subsamples were roughly grouped into three different clusters. According to the bacterial PCoA plot, subsamples from young active sulfate chimney (JDF13 and 14) clustered together, all exterior subsamples of inactive sulfide chimneys (JDF1-3, JDF7, JDF10, and JDF15) formed one close group, and three subsamples JDF4, JDF16, and JDF17 did not cluster with the other samples, and formed loosely clustered group. PCoA appearance of these three subsamples is clearly driven by the highest abundance of the δ*-Proteobacteria* (**Figure 6**). Subsamples collected from middle layers and inner parts of inactive chimneys were distributed among these three distinct groups (**Figure 8**). By contrast, archaeal sequences were grouped into four separate clusters by PCoA (Supplementary Figure S4) and did not form obviously distinguishable clusters according to their spatial positions within the inactive chimney.

## Discussion

In this study, numerous Fe-rich ultrastructures with distinctive morphologies and surficial characteristics were commonly observed from outer parts of an inactive sulfide chimney. These structures were attributed to the combined effect of biogenic oxidation of FeOB and abiotic chemical oxidation. Together with mineralogical results, it appears that the inactive sulfide chimney was experiencing oxidative weathering. Microbial community analysis confirmed the occurrence of well-known FeOB groups in subsamples from the inactive sulfide chimney, *Mariprofundus* spp. within the ζ*-Proteobacteria*, which are common near low-temperature hydrothermal vents and *Gallionella*-associated members generally detected from terrestrial fresh water instead of marine niches. Moreover, although hydrothermal venting had ceased, the inactive sulfide chimney appears to still accommodate diverse microbial populations.

### Oxidative Weathering of Inactive Sulfide Chimneys and Involved Fe-Oxidizing Bacterial Groups

Oxidative weathering of hydrothermal inactive sulfide structures and deposits is prevalent on the modern seafloor. As the result of high-temperature hydrothermal activities, a variety of sulfide minerals are precipitated and further develop into chimney-like structures and massive sulfide deposits that are highly enriched in various reduced forms of metals, including sulfur as well as other elements. Once hydrothermal activities cease, these reduced compounds are exposed to low-temperature, oxygenated seawater, resulting in oxidative weathering reactions (Edwards et al., 2005). The products of seafloor oxidative weathering of sulfide minerals include secondary hydrous alteration minerals, including a variety of Fe oxyhydroxides or oxides together with a few clay minerals, jarosite and atacamite (Pichler et al., 1999; Rogers et al., 2003; Edwards et al., 2004). In some cases, weathered mineral assemblages accumulate extensively on top of massive sulfide deposits as crusts or caps up to several tens of centimeters thick to form gossans (Hannington, 1993; Herzig and Hannington, 1995). In the present study, evidence is seen of oxidative weathering on an inactive sulfide chimney. On the surface, the inactive chimney walls displayed obvious corrosion features and were commonly covered by yellow to reddish-brown alteration products (**Figure 1** and Supplementary Figure S2). These products also filled the fractures, pits, pores and void spaces in the chimney walls. Analysis of mineral components of these alteration products based on XRD and FT-IR determinations (**Figure 2**), together with EDS analyses revealed that they are primarily composed of Fe and O (**Figures 4, 5**), indicative of Fe oxyhydroxides/oxides.

The prevalence of FeOB-associated ultrastructures suggests that Fe-oxidizing species played an important role in oxidative weathering of inactive hydrothermal chimney. Textural observations of altered chimney surfaces revealed that they consisted of Fe-rich twisted or branched stalks, tubular sheaths and contorted filaments (**Figure 4**). Based on their distinctive morphologies and compositions, these ultrastructures closely resemble the biogenic Fe oxides of several known FeOB species, including *M. ferrooxydans, G. ferruginea*, and *L. ochracea* (Emerson et al., 2010; McAllister et al., 2011). It has been shown that Fe oxidizers can directly use different sulfide mineral substrates and actively facilitate the weathering processes (McCollom, 2000; Edwards et al., 2003a,b). Generally, these Fe-oxidizing species are strictly microaerophilic, and hence, it appears that the well-oxygenated seawater that surrounds inactive chimneys does not support their growth. In fact, O_2_-utilizing populations initially colonize the surfaces of inactive chimneys (Edwards et al., 2003a; Rogers et al., 2003) and reduce surface oxygen to levels that FeOB species favor such that they can effectively compete with rapid abiotic Fe^2+^ oxidation. Once Fe oxidizers inhabit the surface and their Fe-oxide crusts are produced, they are partially protected from the oxidative exchanges with seawater (Edwards et al., 2005). In addition, the oxidation of Fe and S is estimated to supply the most abundant energy sources for chemolithoautotrophs in seafloor environments because of their high abundances in basalt and metal sulfides (Bach and Edwards, 2003; Edwards et al., 2005), which, to a large extent, explains the wide distribution of FeOB species within the alteration products of seafloor rocks and sulfide deposits in the ocean crust (Perfit et al., 2003; Rogers et al., 2003; Orcutt et al., 2011).

Fe-oxidizing bacteria-involved biomineralization also commonly occurs within the tubes of dead tubeworms. It has been demonstrated that near hydrothermal vents, tubeworms can remarkably influence metal distribution, and that sulfide minerals (especially iron sulfides) accumulate on surfaces or are entombed within the tube interlayers during tube-building episodes (Maginn et al., 2002; Peng et al., 2009). With the process of mineralization, certain tubes are gradually replaced by pyrite and marcasite and become completely fossilized (e.g., tube T3-1, **Figure 5**). When these tube structures are exposed to seawater, oxidative alterations of various Fe-rich sulfide minerals prevail. Abundant FeOB-associated ultrastructures were also commonly detected on these tubeworm tubes (**Figure 5**).

However, as noted in **Figure 5**, a series of delicate Fe-rich surface textures such as tiny grains, fine burrs and thin nanosheets on the exteriors of FeOB-related ultrastructures of mineralized tubeworm tubes (**Figures 5c,h,l,n**), which are ascribed to the rapid abiotic oxidation of Fe^2+^ instead of microbial activity, indicates that an environmental shift occurred within the tubes from the FeOB-favorable microoxic condition to fully oxygenated niches. Generally, within chimney walls, tube structures of tubeworms create ample fluid conduits through which inward penetration of seawater and outward percolation of dissolved substances within the chimney (hydrothermal fluids are also included) cause intensive and dynamic mixing and further influence the thermal and chemical gradients within the chimney walls (Koski et al., 1984; Cook and Stakes, 1995). Tube T1, which was divided into yellow and gray portions (**Figure 5**), provides an example of the outward transportation of dissolved substances of the chimney. In the yellow portion, Fe oxyhydroxides consisting of FeOB-associated ultrastructures are abundant, whereas they are seldom found in the gray portion. The boundary between yellow and gray roughly indicates the positions at which Fe^2+^ was dissolved and extracted from sulfide minerals by iron oxidizers colonizing the tube surface. A similar situation occurs on the exterior surfaces of chimney walls, where needle-like and flower-like architectures enwrap the FeOB-associated structures (**Figures 4h,j**) and are also attributed to abiotic Fe^2+^ oxidation. The precipitation and accumulation of Fe-rich substances caused by this type of abiotic Fe^2+^ oxidation may cause these FeOB-associated structures to expand outward (**Figures 4h,j, 5k,n,o**). In certain cases, intensive abiotic Fe precipitation can obscure and modify the original shapes and morphologies of biotic structures, or even submerge them to an extent (**Figure 5i**). The coexistence of fresh FeOB-associated ultrastructures and those modified by Fe^2+^ oxidation (**Figures 4k, 5k**) indicates that the interfaces at which sulfide mineral assemblages of the inactive chimney actively react with seawater are highly dynamic and alterative. Depending on the dissolved O_2_ concentrations, FeOB species and abiotic oxidization alternatively dominate the formation of Fe oxyhydroxides. Recently, a study based on the Fe mineralogy and isotope data also reveal similar dynamic microenvironments in seafloor sulfide-mineral deposits with variable redox conditions causing a dynamic range of Fe transformation pathways (Toner et al., 2016). In addition, nanostructures of Fe oxyhydroxides and oxides are sensitive to changes in environmental conditions (Guo and Barnard, 2013; Zhang et al., 2014). Therefore, distinct characteristic morphologies on the surfaces of FeOB-associated ultrastructures infer considerably heterogeneous microenvironments with different physicochemical natures on the inactive sulfide chimney walls.

Phylogenetic analyses suggest that iron oxidizers dwelling within inactive chimneys consist of members of the genus *Gallionella* and the class ζ*-Proteobacteria*. Tags affiliated with the family *Gallionellaceae* account for approximately 0.3% of the total tags on JDF15 (Supplementary Table S1), and clone libraries of JDF5 contain at least three OTUs that are closely associated with *G. ferruginea* (**Figure 7**). In previous studies, members of the genus *Gallionella* were seldom detected with microbiological evidence from the seafloor environments (Edwards et al., 2003a; McAllister et al., 2011). Although twisted ribbon-like stalks are commonly assigned as the biosignature of the *Gallionella* spp. in biogenic iron oxyhydroxides from hydrothermal fields (Fortin et al., 1998; Kennedy et al., 2003; Edwards et al., 2004), a low occurrence of *Gallionella*-like microbes and the new finding that *M. ferrooxydans* can produce almost identical structures (Emerson and Moyer, 2002) have led researchers to argue that twisted ribbon-like stalks on the seafloor should be attributed to members of ζ*-Proteobacteria* (primarily consisting of *M. ferrooxydans* and its relatives) instead of *Gallionella* spp., which are widely distributed in terrestrial freshwater niches rather than marine environments. Our results, together with several phylotypes closely related to *Gallionella* spp. recently obtained from the low-temperature Fe-rich precipitates (Kato et al., 2009; Li et al., 2012), suggest that *Gallionella* spp. indeed exist in deep-sea hydrothermal vents. More recently, Sylvan et al. (2012) revealed that up to 1% of tag sequences were closely related to *Gallionella* spp. and were one of the most common tags detected from an inactive hydrothermal sulfide chimney. A roughly similar situation also occurs regarding the tubular sheaths that are commonly ascribed to *L. ochracea.* No sequences related to this taxon have yet been recovered from hydrothermal chimneys (Emerson and Ghiorse, 1992; McAllister et al., 2011). In a recent study, it was observed that the tubular sheaths were most likely produced by the members of ζ*-Proteobacteria* instead of *L. ochracea* or other iron-oxidizing β*-Proteobacteria* in the veil-like surface layers of the mats at Loihi Seamount (Fleming et al., 2013). More recently, a series of cultivation showed that this kind of tubular sheaths is indeed produced by ζ*-Proteobacteria* (Barco et al., 2017). Combined with the fact that no *Leptothrix*-related species were detected, and members within ζ*-Proteobacteria* were common (Supplementary Table S1), tubular sheaths within our inactive chimneys (**Figure 4**) are attributed to the products of ζ*-Proteobacteria*. Tags closely related to *M. ferrooxydans* within ζ*-Proteobacteria* were commonly distributed among most of the subsamples. Since its initial discovery (Emerson and Moyer, 2002), *M. ferrooxydans* has primarily been found from various iron oxyhydroxide deposits near the hydrothermal vent, basalt and oceanic crust (Emerson et al., 2010). Several recent studies revealed that *M. ferrooxydans* could also be distributed in near-shore and estuarine environments (McBeth et al., 2011; Dang et al., 2014). Taken together, the findings suggest that although morphologic evidence is frequently used to indicate the activity of Fe oxidizers, and although ultrastructures with different morphologies have been linked phylogenetically to specific FeOB species (Kennedy et al., 2003; Emerson et al., 2010), morphological signatures are at times not reliable for distinguishing certain specific iron-oxidizing species, and true microbiological evidence is still required.

### Microbial Communities Dwelling within Inactive Sulfide Chimneys

Total organic carbon contents of our inactive chimney (0.04–1.91%) are comparable to or even higher than those of active chimneys (**Figure 3**), implying high microbial biomass and active microbial activity within the chimney samples investigated here. It has been suggested that, in general, active hydrothermal structures contain TOC concentrations ranging from 0.02 to 1.0 wt% (Kato et al., 2010; Jaeschke et al., 2012, 2014; Reeves et al., 2014) and occasionally, local patchy areas have high TOC content of nearly 3.0 wt% (Jaeschke et al., 2012). To a certain degree, values of δ^13^C_TOC_ can roughly reflect the chemolithoautotrophic activities in such habitats (Kato et al., 2010; Jaeschke et al., 2014). Several exterior subsamples of the inactive chimney Milli-Q S10 show the lowest δ^13^C_TOC_ values (-31.39 to -29.48‰) with TOC concentrations that are also high (0.13–0.31 wt%) (**Figure 3**), suggesting that in these exterior portions, chemolithoautotrophic activities (for example, FeOB-associated oxidation of chimney minerals) might be higher than those in the middle and interior portions. In this work, we do not exclude the influence of organic remains originating from microbial inhabitants and symbiotic autotrophic lineages of invertebrates within the chimney before the hydrothermal supply ceased. In addition, the δ^13^C_TOC_ values of the inactive chimney are usually low (-31.4 to -21.2‰) compared with those of active chimneys previously reported (-21.4 to -15.5‰), possibly attributed to heterotrophic carbon consumption by heterotrophs (Kato et al., 2010; Reeves et al., 2014). In addition, these strong variations in TOC contents and δ^13^C_TOC_ values within different spatial positions indicate that inactive chimney actually hosts quite heterogeneous habitats for microbes.

Inactive sulfide chimney still accommodates flourishing and diverse microbiomes. When the supply of hydrothermal fluid ceases, environmental conditions across chimneys change, resulting in a corresponding shift in the microbiome. Bacterial populations within the inactive chimney investigated here primarily consist of members of δ- and γ*-Proteobacteria*, together with relatively abundant and varying proportions of members within α*-Proteobacteria, Bacteroidetes, Actinobacteria, Firmicutes*, and *Planctomycetes* (**Figure 6A**). These bacterial components are either seldom detected from active hydrothermal vents or occur but with quite low abundances. For example, most of the tags of *δ-Proteobacteria* were classified into the order *Bdellovibrionales* (Supplementary Table S1), whose members prey exclusively on other bacteria (Schoeffield and Williams, 1990) and are ubiquitously found in various marine environments such as seawater and sediments (Welsh et al., 2015; Yilmaz et al., 2016). Recently, although members of *Bdellovibrionales* were detected from seafloor vents, they occurred only in inactive sulfide chimneys similar to ours (Sylvan et al., 2012) and rather low-temperature (<15°C) “snowblower” hydrothermal vents (Meyer et al., 2013). Within γ*-Proteobacteria*, tags affiliated with the genus *Nitrosococcus* which is known as typical ammonia-oxidizing bacteria (AOB), commonly occur in the inactive chimney with less than 5% proportions, but they are the most abundant bacterial populations in JDF4, in which they were detected in nearly 54% of tags recovered (Supplementary Table S1). Within the subsample JDF4, tags affiliated to the genus *Desulfobulbus* of δ*-Proteobacteria* are also abundant with more than 15% proportions. Members of the *Desulfobulbus* comprise strictly anaerobic, sulfate reducers utilizing sulfate (sulfite or thiosulfate) as the terminal electron acceptor with the oxidation of organic compounds (chemoorganotroph) (Kuever et al., 2015). There is a large number of tags within the genus *Pseudomonas* together with minor members of the genera *Acinetobacter* and *Flavobacterium*. All these groups are putative denitrifiers. Interestingly, these three taxa are abundantly distributed within the same spatial areas within the active chimneys and are the dominant bacterial populations (∼60% of reads) within the relatively inner regions such as JDF11, JDF8, JDF6 and JDF12, and the actively young CAP sulfate chimney. Nitrite oxidizers within the inactive chimneys are mainly represented by two bacterial genera, the genus *Nitrospira* of the phylum *Nitrospira* and the genus *Nitrospina* of δ*-Proteobacteria*. Compared with other participants of N-cycling noted above, their distribution is limited among the chimneys; moreover, their abundance is generally less than 0.5% of total tags, and within the outmost surfaces, their proportion might be near only 1% (Supplementary Table S1). Based on phylogenetic analyses of bacterial communities, it is inferred that they are potentially involved in a diverse set of geochemical processes including iron oxidation (*Gallionella* spp. and *Mariprofundus* spp.), methane oxidation (*Methylococcales* of γ*-Proteobacteria*), nitrogen fixation (e.g., *Rhizobiales* of α*-Proteobacteria*), ammonia oxidation (e.g., *Nitrosococcus* of γ*-Proteobacteria*) and denitrification (*Pseudomonas* of γ*-Proteobacteria*), which indicates that diverse nutrient supplies are available compared with active sulfide chimneys that to a great extent depend on sulfur oxidization (Campbell et al., 2006; Kormas et al., 2006; Li et al., 2014). A similar situation occurs with archaeal community structures. In three subsamples of inactive chimney material no archaeal tags were detected successfully, suggesting the low biomass and restricted distribution of archaea within inactive chimney. By contrast, in active hydrothermal chimneys, archaea are generally abundant (Schrenk et al., 2003; Kormas et al., 2006; Li et al., 2014). Mesophilic archaea, for example, members of the *Thaumarchaeota*, are abundant within the inactive chimney, even in their middle and inner parts (**Figure 6B**), which suggests decreasing temperatures within inactive chimneys as high-temperature fluid supply stops. Inhabitants of inactive chimney, especially bacterial and thaumarchaeotal taxa, may start to largely reflect normal marine seawater and sediment communities as venting ceases. In the process of colonization from surrounding seawater and sediments, the mineralogy and degree of exposure to seawater likely selects for specific bacterial communities (Edwards et al., 2003a; Toner et al., 2013). This notion is supported by the observation that taxonomic signatures of bacteria within most samples from the outer surface of the inactive chimney cluster together (**Figure 8**).

The detection of diverse archaeal communities within the inactive chimney, especially the prevalence of thermophilic and hyperthermophilic taxa (**Figure 6B**), was unexpected. Previously, archaeal populations were generally absent from inactive chimneys, indicating archaea might not be important in the weathering of minerals and rocks on the seafloor relative to bacteria (Edwards et al., 2003a; Rogers et al., 2003; Kato et al., 2010; Sylvan et al., 2012; Reeves et al., 2014). Recently archaeal taxa were shown to represent only a small proportion of the microbiome in inactive chimneys (below detection or <0.2%) (Sylvan et al., 2013). Although archaeal populations appeared within our inactive chimney subsamples, there were a couple of inactive chimney subsamples from which no archaeal tags were obtained, indicating archaeal signatures were likely in low abundance. When hydrothermal activities cease, inactive chimney walls are surrounded by low-temperature seawater, conditions not favorable for thermophiles. Two possibilities may explain the detection of signatures of thermophiles. One is that the supply of high-temperature fluids ceased only recently, and these thermophilic DNA signatures are the remains of previous archaeal colonizers that inhabited the hot active chimney. Another possibility is that these thermophiles are sourced from sub-seafloor hydrothermal habitats and are subsequently transported into inactive chimneys within periodic pulses of low-temperature fluid from below the ridge flank. Recently, new findings suggest that ecologically unique high-temperature archaeal reservoirs exist beneath ridge flanks (Kormas et al., 2003; Ehrhardt et al., 2007; Edwards et al., 2011) in which crustal fluids support these active indigenous communities (Cowen et al., 2003; Huber et al., 2006; Engelen et al., 2008). PCoA analysis of archaea shows that subsamples were not separated by their spatial zonation (Supplementary Figure S4), especially exterior samples. This supports to some degree the possibility that they come from deep beneath the seafloor and are randomly distributed within the inactive chimney.

### Microbial Colonization within the Young Sulfate Hydrothermal Chimney and Microbial Succession during Chimney Evolution

Microbial communities within the CAP sulfate chimney reflect early colonizers during the formation of a hydrothermal chimney. As noted above, the CAP chimney walls are porous and primarily composed of anhydrite (**Table 1**). The mineralogical compositions, i.e., the absolute dominances of sulfate minerals, indicate that the CAP chimney is still in the young first formation stage (Tivey, 1995), although its exact age was not determined in the present study. *In situ* growth experiments that simulate chimney formation showed that young hydrothermal chimneys with sulfate mineral assemblages that are roughly similar to the current CAP chimney could be quickly deposited and well-developed within several days to months (Page et al., 2008; Wang F. et al., 2009). Our microbial analyses revealed that microbial signatures associated with this chimney were dominated by members of γ*-Proteobacteria*, followed by *Bacteroidetes* and the *Firmicutes*, and no archaeal components were detected (**Figure 6A** and Supplementary Table S1). Heterotrophic bacterial populations, including members of the orders *Pseudomonadales, Flavobacteriales*, and *Lactobacillales* represent more than 85% of sequenced tags (Supplementary Table S1). This contrasts with previous observations showing the general prevalence of autotrophic 𝜀*-Proteobacteria* (Reysenbach et al., 2000; López-García et al., 2003; Alain et al., 2004). The most likely explanation for the dominance of heterotrophs is that a rapid shift has occurred from autotrophs to heterotrophs within this young sulfate chimney. As primary producers, chemolithoautotrophs are likely the first colonizers to occupy exposed surfaces within sulfate chimneys. Once sufficient organic carbon accumulates, heterotrophic bacterial lineages may emerge and replace the autotrophs as the dominant taxa (Reysenbach et al., 2000). Variation in microbial succession can occur within hours, days, or months (Reysenbach et al., 2000; Nercessian et al., 2003; Page et al., 2008). In addition, the absence of archaeal clades from this nascent chimney is consistent with previous *in situ* colonization observations (López-García et al., 2003). *In situ* temperature is expected to be one of the crucial constraints on microbial colonization (Alain et al., 2004). Most archaeal lineages near hydrothermal vents generally consist of thermophiles or hyperthermophiles that inhabit the hottest areas of the chimney. Although the CAP chimney is active, the temperature within most positions of the sulfate chimney walls [especially in the middle and outer regions where we sampled (**Figure 1c**)] is inferred to be low, which is supported by the lack of high-temperature sulfide mineral assemblages (**Table 1**). Bacterial signatures from the middle (JDF14) and outer portions (JDF13) are not obviously distinct, indicating their possibly homologous environmental parameters.

**FIGURE 9:**
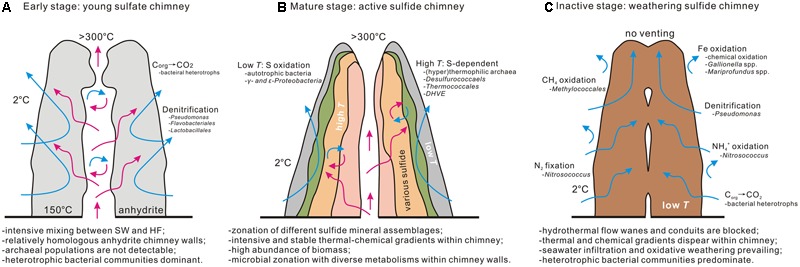
Conceptual model of microbial succession at different stages of hydrothermal chimney evolution. The characteristics of physical–chemical conditions, microbial population, and involved geochemical processes are included. The data of early stage **(A)** and inactive stage **(C)** come from our present study and mature stage **(B)** depends mainly on previous work (Li et al., 2014).

Combining microbial communities that inhabit the young CAP sulfate chimney and the inactive sulfide chimney in the current study with those in maturely active sulfide structures collected from the same MEF hydrothermal field in previous investigations (Zhou et al., 2009; Li et al., 2014) provides a rough depiction of the microbial community succession at different stages of hydrothermal chimney aging (**Figure 9**). At the early nascent stage, the chimney is precipitated initially and characterized by the dominant anhydrites. Microbial communities appear to be mainly dominated by heterotrophic bacterial populations, including members of γ*-Proteobacteria* together with relatively abundant members of *Bacteroidetes* and *Firmicutes*. The abundance of archaeal communities is not high. Moreover, archaeal populations are generally absent from the chimney at this stage because of unfavorable environmental conditions, including low temperatures and high concentrations of oxygen. With the gradual precipitation of diverse sulfide minerals, the mature chimney is solidified and dominated by high-temperature mineral assemblages. Correspondingly, the microbial community structure changes dramatically. Bacterial compositions primarily consist of autotrophic members of 𝜀*-Proteobacteria*, whereas archaeal members are dominated by thermophiles and hyperthermophiles generally involved in sulfur cycling (Zhou et al., 2009; Li et al., 2014). Microbial abundance and taxonomic composition varies significantly along the profile of an active chimney from the outer surfaces to the inner regions close to the central hydrothermal conduits (Li et al., 2014). Finally, with waning and cessation of hydrothermal activity, energy supporting the microbial populations changes from the extensive chemical disequilibrium between seawater and hydrothermal fluid to sulfide weathering and decomposition of organic remains. At this stage, bacterial communities appear dominated by members of γ*-Proteobacteria*, followed by *Bacteroidetes* and *Firmicutes*. These bacterial clades primarily consume pre-formed organic materials. Where Fe-rich sulfide minerals interact with seawater, autotrophic FeOB appear to flourish. At the same time, archaeal members gradually disappear with the waning of hydrothermal supplies, and certain mesophilic archaeal components such as *Thaumarchaeota* remain. However, in selected situations, pulses of low-temperature fluids may carry thermophilic and hyperthermophilic species derived from deep niches into inactive chimneys.

## Conclusion

In this study, the oxidative weathering and microbial diversity of an inactive hydrothermal sulfide chimney and a young, active chimney were analyzed. Oxidative weathering commonly occurred on the inactive sulfide chimney. Fe oxidizers closely related to *Gallionella* spp. and *Mariprofundus* spp. appear to promote this process based on abundant remains of distinctive Fe-rich ultrastructures. At the same time, a series of characteristic tissues encrusted on the surface of FeOB-associated ultrastructures and ascribed to abiotic Fe^2+^ oxidization are distinguished, and their coexistence indicates that oxidative weathering is a dynamic process with environmental parameters that are continuously varying. Phylogenetic analysis of pyrotag sequences suggests that inactive sulfide chimneys can still accommodate abundant and diverse microbial populations even though the hydrothermal supply has ceased, but composition and inferred metabolic potential significantly differ from those of an active high-temperature sulfide chimney.

## Author Contributions

JL and HZ designed the research. JL and QY subsampled the chimneys. JC, BW, and ZW conducted electron microscopic analyses, mineralogical contents and TOC determinations. JL, GC, and YW performed molecular diversity analyses. JL and HZ wrote the manuscript. All authors contributed to interpretation of data.

## Conflict of Interest Statement

The authors declare that the research was conducted in the absence of any commercial or financial relationships that could be construed as a potential conflict of interest.
