# Supplementary material for: Oxidative Weathering and Microbial Diversity of an Inactive Seafloor Hydrothermal Sulfide Chimney
Source: Front Microbiol. 2017 Jul 21;8:1378. doi: 10.3389/fmicb.2017.01378 (PMC5519607; doi:10.3389/fmicb.2017.01378)
Supplement: Supplementary file 3 [file Data_Sheet_1.DOCX]

Supplementary Material

Oxidative weathering and microbial diversity of an inactive seafloor hydrothermal sulfide chimney

Jiangtao Li, Jiamei Cui, Qunhui Yang, Guojie Cui, Bingbing Wei, Zijun Wu, Yong Wang and Huaiyang Zhou*

*** Correspondence:** Huaiyang Zhou: zhouhy@tongji.edu.cn

# 1. Supplementary Figures and Tables

## 1.1 Supplementary Figures


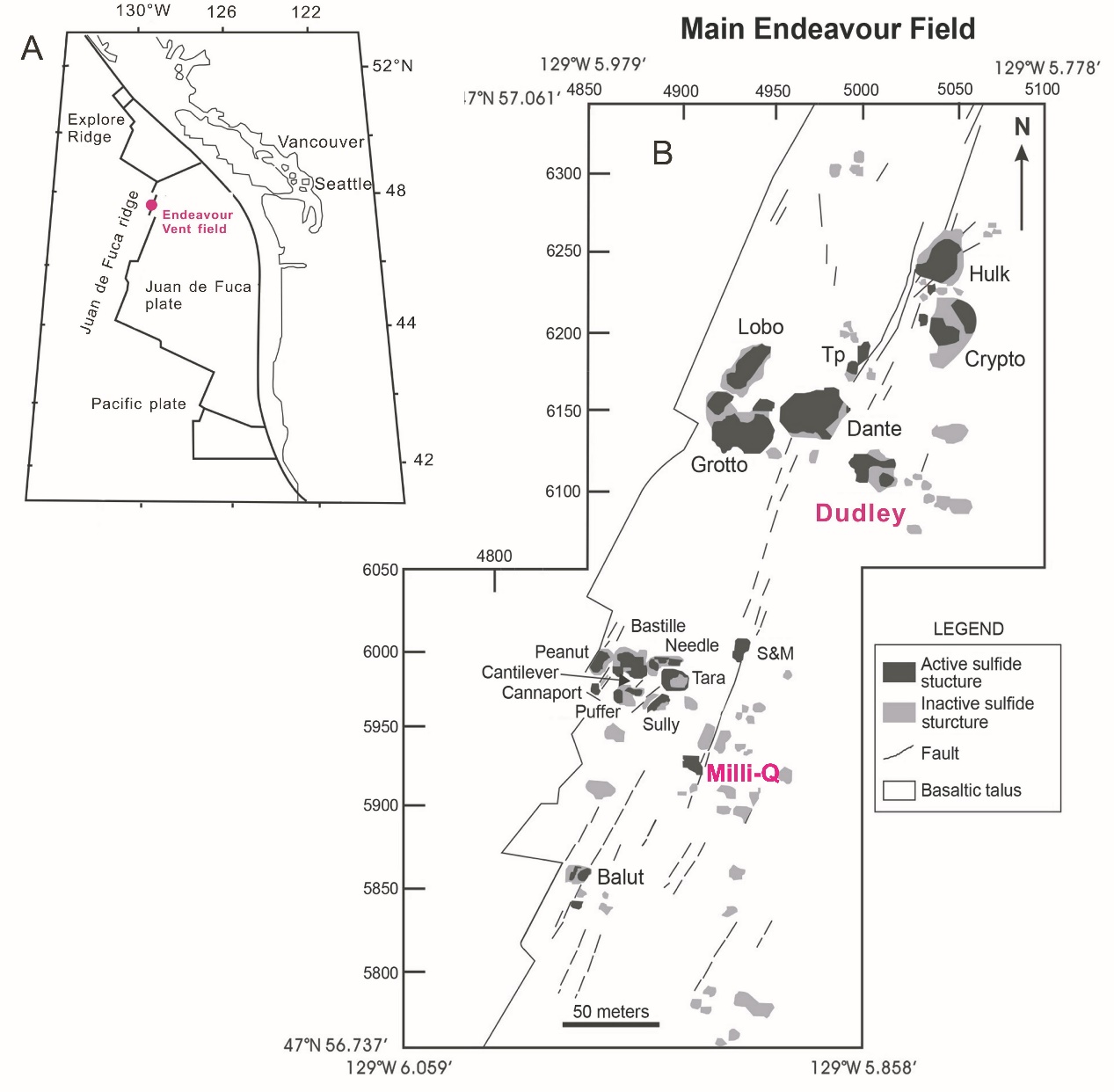


**Supplementary Figure S1**. Sampling locations of the Milli-Q and Dudley hydrothermal vent sites (B) in Main Endeavour Field of the Juan de Fuca Ridge (A) (modified after Delaney et al., 1992; Butterfield et al., 1994).


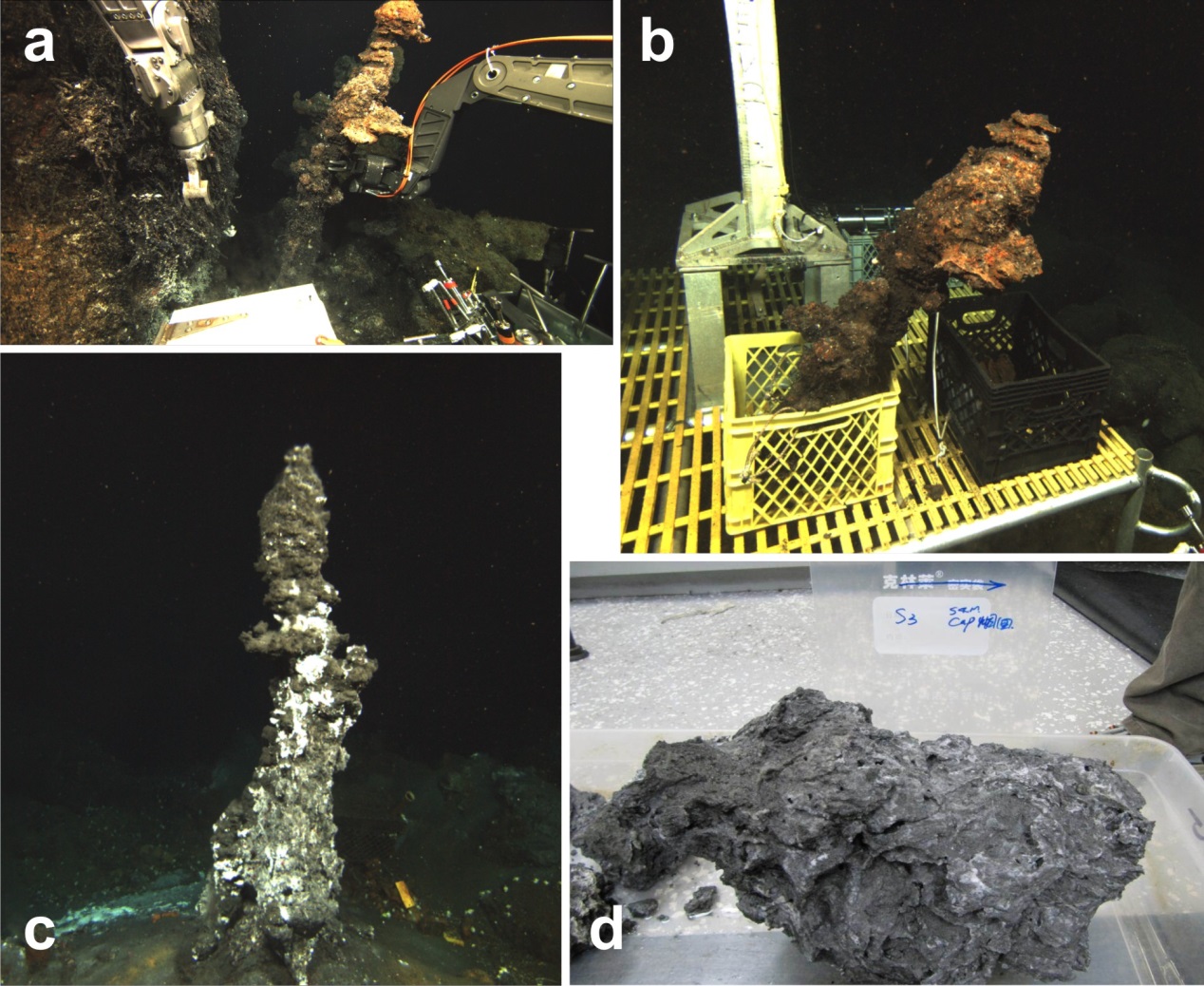


**Supplementary Figure S2**. **(a, b)** Sampling of inactive sulfide chimney by ROV Jason II from the seafloor of Milli-Q site. **(c, d)** Immature sulfate chimney (CAP) in the seafloor of Dudley site and the chimney shipped to the lab.


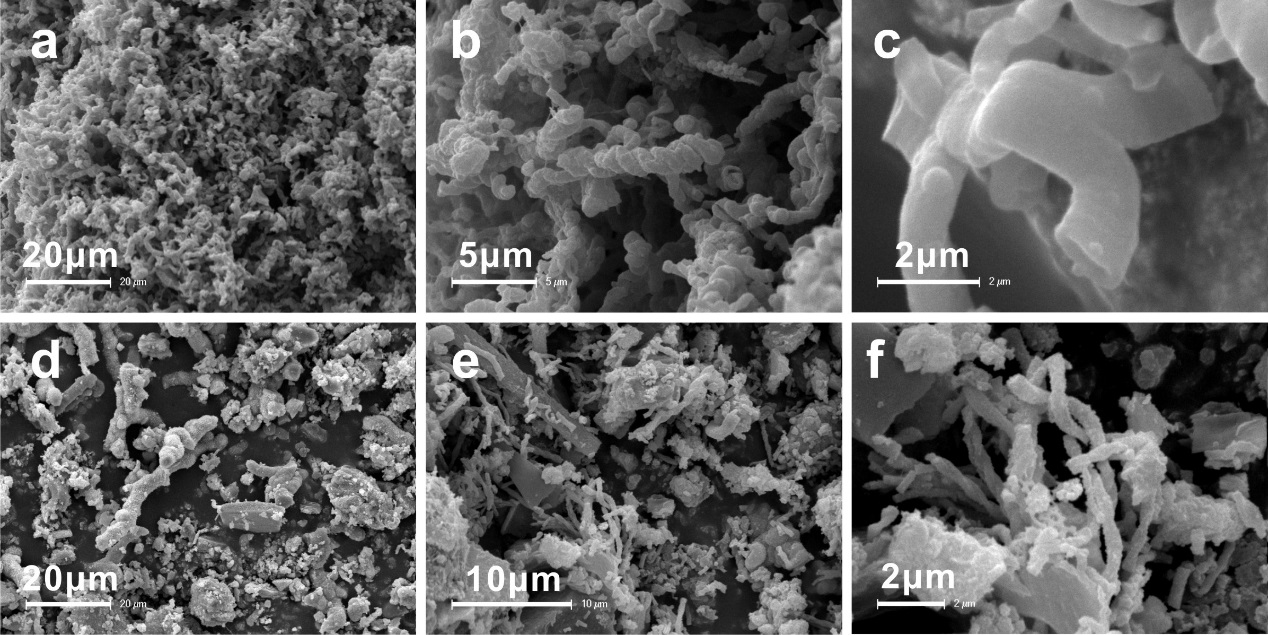


**Supplementary Figure S3.** SEM images of typical ultrastructures from the JDF5 microbial mats. **(a)** Dense FeOB-associated ultrstructures including branched stalks, twisted stalks and filaments. **(b)** Typical twisted stalks encrusted by Fe-rich substances. **(c)** Fresh hollow, branched Fe-rich stalks. **(d)** Ttwisted stalks heavily coated by acanthaceous Fe-rich aggregates. **(e)** various Fe-rich biogenic ultrastructures and abiotic aggregates. **(f)** Magnification of panel (e) showing twisted ribbon-like stalks were attached by formless Fe-rich aggregates.


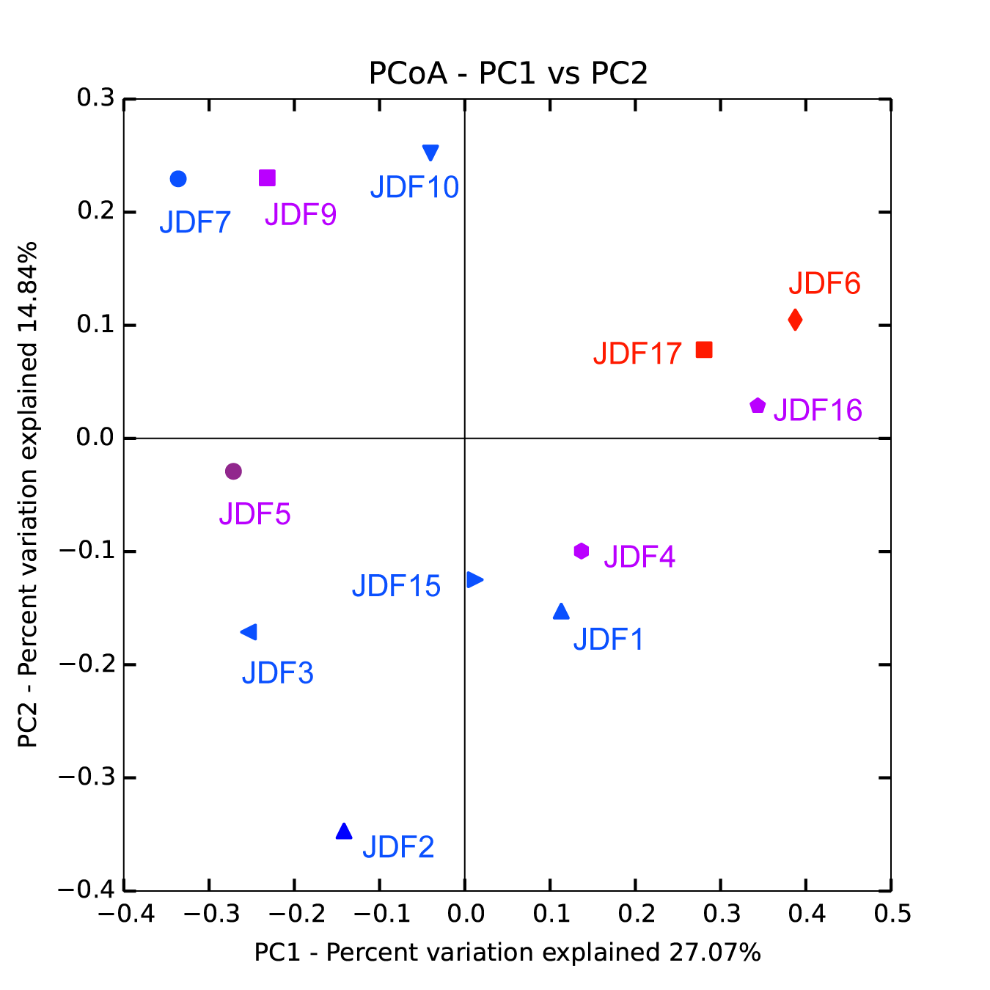


**Supplementary Figure S4.** Principal component analysis (PCoA) of archaeal 16S rRNA gene sequences obtained from different spatical positions of inactive sulfide chimney. ***Red:*** subsamples from inner parts; ***Purple:*** subsamples from middle layers; ***Blue:*** subsamples from exterior walls.

## 1.2 Supplementary Tables

**Supplementary Table S1.** Bacterial compositions at different taxon levels

**Supplementary Table S2.** Archaeal compositions at different taxon levels
